# Cognitive Behavioral Therapy vs Media Literacy for Prevention of Gaming Disorder and Unspecified Internet Use Disorder: A Randomized Clinical Trial

**DOI:** 10.1001/jamanetworkopen.2026.31942

**Published:** 2026-09-04

**Authors:** Katajun Lindenberg, Sonja Kewitz, Ina Neumann, Sebastian Brand, Daniel Huth, Julia Lardinoix, Katharina Leo, Carolin Szász-Janocha, Daniel L. King, Lutz Wartberg

**Affiliations:** 1 Department of Clinical Psychology of Childhood and Adolescence, Institute for Psychology, Heidelberg University, Heidelberg, Germany; 2 Department of Psychotherapy for Children and Adolescents, Institute for Psychology, Goethe University Frankfurt, Frankfurt am Main, Germany; 3 Department of Clinical Psychology and Psychotherapy for Childhood and Adolescence, Johannes Gutenberg-University Mainz, Germany; 4 College of Education, Psychology and Social Work, Flinders University, Adelaide, Australia; 5 Department of Psychology, Faculty of Human Sciences, MSH Medical School Hamburg, Hamburg, Germany

## Abstract

**Importance:**

Mitigating the harmful effects of digitalization on youth mental health is essential.

**Objectives:**

To evaluate the effectiveness of cognitive behavioral therapy (CBT) compared with a media literacy–based intervention for gaming disorder and unspecified internet use disorder and to determine whether universal or indicated prevention yields stronger benefits.

**Design, Setting, and Participants:**

PROTECTconfirm was a randomized clinical trial conducted from July 1, 2020, to August 31, 2024, across 44 secondary schools in Baden-Württemberg, Germany, with 1-, 4-, and 12-month follow-up. All students were included in universal prevention analyses, whereas indicated prevention analyses included a subgroup of adolescents at high risk meeting 2 or more *Diagnostic and Statistical Manual of Mental Disorders* (Fifth Edition) criteria for gaming disorder or unspecified internet use disorder at baseline.

**Interventions:**

Students were individually randomized within 90 classes to PROTECTtraining, a CBT-based program; or PROTECTinfo, a structurally parallel media literacy program. Both consisted of four 90-minute sessions delivered weekly during school hours by trained local prevention professionals under live supervision and adherence protocols.

**Main Outcomes and Measures:**

The primary outcome was gaming disorder symptom severity and unspecified internet use disorder symptom severity, assessed at 12 months with a modified version of the Video Game Dependency Scale. Secondary outcomes included internalizing, externalizing, and transdiagnostic symptoms. Primary and secondary outcomes were analyzed according to the intention-to-treat principle.

**Results:**

A total of 1793 students were randomized to PROTECTtraining (n = 896; mean [SD] age, 13.1 [1.5] years; 498 male students [56.1%]) or PROTECTinfo (n = 897; mean [SD] age, 13.1 [1.5] years; 484 male students [54.8%]). Participants in the subsample of 205 adolescents at high risk showed significantly lower 12-month severity of gaming disorder and unspecified internet use disorder symptoms with PROTECTtraining than with PROTECTinfo (mean [SD] Modified Video Game Dependency Scale score: 14.7 [9.7] vs 17.7 [10.3]; within-group Cohen *d* = −0.91 [95% CI, −1.10 to −0.72] vs Cohen *d* = −0.61 [95% CI, −0.80 to −0.42]; between-group Cohen *d* = −0.30 [95% CI, −0.55 to −0.05]), but not in the total sample (mean [SD] Modified Video Game Dependency Scale score: PROTECTtraining, 8.8 [8.3] vs PROTECTinfo, 9.4 [8.4]; within-group Cohen *d* = −0.11 [95% CI, −0.18 to −0.05] vs Cohen *d* = −0.04 [95% CI, −0.11 to 0.02]; between-group Cohen *d* = −0.07 [95% CI, −0.16 to 0.01]). Secondary outcomes did not differ significantly between groups.

**Conclusions and Relevance:**

In this randomized clinical trial of 1793 adolescents, the CBT-based intervention reduced gaming disorder symptoms and unspecified internet use disorder symptoms more effectively than the media literacy–based intervention among adolescents at high risk. These findings support the use of CBT-based approaches primarily within indicated, rather than universal, prevention.

**Trial Registration:**

German Clinical Trials Register (DRKS) trial registration: DRKS00033989

## Key Points

**Question:**

Is cognitive behavioral therapy more effective than media literacy in reducing symptoms of gaming disorder and unspecified internet use disorder?

**Findings:**

In this randomized clinical trial of 1793 adolescents, cognitive behavioral therapy–based prevention produced significantly lower 12-month severity of symptoms of gaming disorder and unspecified internet use disorder than media literacy in a subsample of adolescents at high risk (mean Modified Video Game Dependency Scale score, 14.7 for cognitive behavioral therapy vs 17.7 for media literacy), but not in the total sample (mean Modified Video Game Dependency Scale score, 8.8 for cognitive behavioral therapy vs 9.4 for media literacy).

**Meaning:**

These findings suggest that cognitive behavioral therapy–based prevention of gaming disorder and unspecified internet use disorder is more effective than media literacy–based prevention when targeted at adolescents at high risk.

## Teaser

This randomized clinical trial compares the effectiveness of cognitive behavioral therapy with a media literacy–based intervention in reducing symptoms of gaming disorder and unspecified internet use disorder among adolescents.

## Introduction

With the inclusion of gaming disorder as a formal diagnosis in the *International Classification of Diseases, 11th Revision* (*ICD-11*),^1^ behavioral addictions have gained international recognition as significant public health challenges. Clinically relevant misuse of online activities, such as gambling, gaming, social media use, shopping, or pornography, has been conceptualized as *internet use disorder* (as an umbrella term), which involves substantial functional impairment, psychological distress, and social or academic difficulties.^2,3,4,5^ Adolescents represent a particularly vulnerable population due to ongoing neurodevelopment, heightened reward sensitivity, and evolving self-regulation capacities, which increase susceptibility to compulsive and reward-driven patterns of digital media use.^6^

Epidemiologic studies consistently show that problematic use of the internet affects a substantial proportion of adolescents worldwide, with estimates for subclinical problematic behaviors ranging up to 25%, depending on diagnostic criteria and assessment methods.^2^ Meta-analyses estimate the global prevalence of gaming disorder to be 2% to 3%^7,8^ and of problematic social media use to be 5%.^9^ The COVID-19 pandemic created conditions that increased the risk for behavioral addictions, including social isolation, reduced physical activity, and greater availability and attractiveness of online offers, thereby potentially reinforcing problematic use patterns.^10^ This underlines the need for evidence-based prevention strategies targeting maladaptive patterns of online behavior during adolescence.^11^

School-based prevention is regarded as a key setting for mitigating the harmful effects of digitalization on youth mental health^12^ and is recommended in the German clinical guidelines on early interventions for internet use disorders.^13^ To mitigate the potentially harmful effects of digitalization on youth mental health, some educational systems have implemented media literacy (ML) programs that focus on improving knowledge and digital competencies. However, there is little empirical evidence that such programs effectively reduce symptoms of behavioral addictions or prevent related mental health problems. In contrast, cognitive behavioral therapy (CBT)–based interventions are well studied as a treatment for behavioral addictions^14^ and several studies have also demonstrated their efficacy in preventive settings.^13^ CBT interventions directly target mental mechanisms known to contribute to internet use disorders,^5^ such as maladaptive behavioral coping, dysfunctional cognitions, and deficits in emotion regulation and impulse control.^15,16,17^

A prior efficacy trial^18^ demonstrated that the CBT-based PROTECT (Professioneller Umgang mit technischen Medien [Professional Use of Technical Media])^15^ intervention reduced symptoms among adolescents at high risk compared with an assessment-only control condition. However, the effectiveness of PROTECT under routine school conditions and in universal samples remains unclear.

Indicated prevention may yield larger effects but requires screening, whereas universal prevention is easier to implement but may produce smaller effects.^12,19,20,21^ To date, data directly comparing these preventive approaches are scarce. Evaluating whether a CBT-based prevention program remains effective under routine school conditions and within universal samples, including subgroup analyses by risk status, therefore represents a translational step.

Furthermore, to test the specificity of CBT-related effects, comparison with an active control condition such as an ML-based training is important. This distinction allows differentiation between general program and person effects (eg, attention effects, group interaction) and mechanisms specifically linked with cognitive or behavioral change. In addition, the quality of program implementation, including adherence to the manual and the quality of the facilitator-participant relationship may be critical for intervention effectiveness and should be evaluated systematically.

The PROTECTconfirm study was designed to confirm the effectiveness of the CBT-based PROTECT program^15,18^ to reduce symptoms of adolescent gaming disorder and unspecified internet use disorder under routine conditions and evaluate its superiority against a placebo control intervention (ie, a structurally parallel ML-based intervention), as well as to evaluate differential effects between universal and indicated prevention populations in a large randomized clinical trial.

## Methods

### Trial Design

PROTECTconfirm was a multicenter, 2-arm randomized superiority trial conducted from July 1, 2020, to August 31, 2024, in 44 secondary schools in Baden-Württemberg, Germany, under routine school conditions. The trial compared a manualized CBT-based program (PROTECTtraining) with a structurally parallel ML-based program (PROTECTinfo). Both interventions were developed without participant or public involvement. All participants were assessed at baseline, 1-month follow-up, 4-month follow-up, and 12-month follow-up. The study was approved by the Ethics Committee of Goethe University Frankfurt and authorized by the Ministry of Education of Baden-Württemberg. Reporting of this study followed the 2025 Consolidated Standards of Reporting Trials (CONSORT) reporting guideline^22^ and the Template for Intervention Description and Replication (TIDieR) guideline.^23^ The trial was conducted in accordance with the Declaration of Helsinki^24^ and EU data protection regulations. Written informed consent from parents and written assent from adolescents were obtained prior to participation. The study was registered in the German Clinical Trials Register (DRKS00033989).

Two additional scales (PBS [Pathological Buying Screener] and PPCS-6 [Problematic Pornography Consumption Scale-Short Version]) were added after data collection began but prior to trial registration to assess problematic shopping and pornography use, given their increasing relevance to problematic use of the internet during adolescence. The amendment was approved by the ethics committee and did not affect primary outcomes or analyses. No other changes to the trial protocol, outcomes, or analyses were made. Full protocol details are provided in Supplement 1. Further details on trial design are available in the eMethods in Supplement 2.

### Participants and Setting

Participants were students aged 11 to 18 years recruited from 92 classes across 44 secondary schools that were representative across school levels (academic track, intermediate track, and lower track) and regions (rural and urban) in the federal state of Baden-Württemberg, Germany. Recruitment occurred from September 1, 2020, to July 31, 2023, with follow-up through July 31, 2024. Project schools incorporated the interventions into their regular curriculum and all students in participating classes received either PROTECTtraining or PROTECTinfo in half-class groups. However, participation in the effectiveness study was voluntary and required informed consent. Only students for whom consent was provided participated in the repeated assessments and contributed data to the study. Of 2198 students from 92 classes receiving the interventions, 1793 from 90 classes (81.6%) participated in the study. Two classes received the interventions but did not participate in data collection for organizational reasons. Interventions and assessments were conducted during regular school hours and onsite, with the exception of 4 classes during COVID-19–related school closures, which received the interventions and assessments online. For allocation of students to the planned universal vs indicated prevention analyses, risk status was classified at baseline using a modified version of the Video Game Dependency Scale (CSAS [Computerspielabhängigkeitsskala]).^25^ Adolescents meeting 2 or more *Diagnostic and Statistical Manual of Mental Disorders* (Fifth Edition) (*DSM-5*) criteria for gaming disorder or unspecified internet use disorder were classified as high risk for indicated analyses, while universal analyses included all participants (eMethods in Supplement 2).

### Randomization and Masking

After consent, students were individually randomized within classes (1:1 ratio) using a prespecified alternating sequence stratified by class, with random starting condition. The allocation sequence was generated by investigators (K. Lindenberg and S.K.) and concealed from enrolling personnel, including teachers (see eMethods in Supplement 2 for details on group allocation). Owing to the nature of the interventions, participants and facilitators were not blinded to allocation but were unaware that the ML-based condition served as the control group and of the study’s superiority hypothesis.

### Interventions

Both manualized programs consisted of 4 weekly 90-minute sessions delivered during school hours by trained local prevention professionals (eg, social workers, school psychologists, teachers, youth counselors). Facilitators completed standardized 4-day training workshops and delivered the interventions under live supervision by expert psychologists, who conducted standardized fidelity ratings to monitor protocol adherence.

#### Intervention 1 (PROTECTtraining)

PROTECTtraining^18^ is a CBT-based intervention targeting motivation to change, maladaptive cognitions, dysfunctional coping, and deficits in emotion regulation. Core components include psychoeducation on addictive mechanisms, cognitive restructuring, behavioral activation, problem solving, and emotion regulation skills training.

#### Intervention 2 (PROTECTinfo)

PROTECTinfo is an ML-based intervention matched with PROTECTtraining in format and dose. It addresses smartphone use, privacy settings, online communication, and risks and benefits of video gaming, as well as cyberbullying and legal aspects of digital behavior. It does not include cognitive behavioral skills training. Further intervention details are provided in the eMethods in Supplement 2.

### Primary and Secondary End Points

The primary outcome was self-reported severity of gaming disorder or unspecified internet use disorder symptoms at 12-month follow-up, assessed with the CSAS, which has 18 items on a 5-point Likert scale, where higher scores indicate greater severity of gaming disorder or unspecified internet use disorder symptoms.^18,25,26^ Unspecified internet use disorders (*ICD-11 *code 6C5Z) capture the 3 categories (ie, problematic pornography use, buying or shopping, social media use) for other specified disorders due to addictive behaviors (*ICD-11 *code 6C5Y)^4^ plus problematic streaming. We adapted the CSAS items to cover both gaming disorder and unspecified internet use disorder in a common score (eg, item 1: “Even when I am not gaming/online, I think about online gaming/the internet” for preoccupation) with permission by the publisher.

Secondary outcomes included general internet use disorder symptoms (short CIUS [Compulsive Internet Use Scale]^27^), problematic gaming (GAT [Gaming Attitude Test],^28^ IGD-9 [9-item Internet Gaming Disorder Scale],^29^ GDT [Gaming Disorder Test]^30^), problematic social media use (SMDS [Social Media Disorder Scale]^31^), general psychopathology (SDQ [Strengths and Difficulties Questionnaire]^32^), attention-deficit/hyperactivity disorder (ADHD; Conners-3 Index^33^), depression (DesTeen [Depression Screener for Teenagers]^34^), social anxiety (SIAS [Social Interaction Anxiety Scale]^35^), performance anxiety (FSSC-R [Fear Survey Schedule for Children–Revised]^36^), emotion regulation difficulties (DERS-SF [Difficulties in Emotion Regulation Scale Short Form]^37^), procrastination (PFS [Procrastination Questionnaire for Students]^38^), quality of life (KIDSCREEN-27^39^), and well-being (WHO-5 [5-item World Health Organization Well-Being Index]^40^). Treatment fidelity was rated by supervisors for each session using standardized protocols. Sociodemographic characteristics, academic performance, and personality traits (BFI-K KJ [Kurzversion des Big Five Inventory für Kinder und Jugendliche]^41^) were assessed at baseline and satisfaction (student evaluation and multiplier evaluation) was assessed after the intervention. Detailed descriptions of all outcome measures and additional measures on problematic gambling,^42^ pornography use,^43^ shopping,^44^ alcohol use,^45^ and cannabis use^46^ are provided in the eMethods and eTable 1 in Supplement 2.

### Harms and Concomitant Care

Potential harms were defined as unintended reliable deterioration in the primary outcome as assessed by the Reliable Change Index from baseline to 12 months of a score of 1.96 or higher. Concomitant care received during the trial was not assessed due to the preventive nature of the trial design.

### Sample Size

Sample size estimation assumed a small effect (Cohen *d* = 0.20), α = .05, and a power of 80%, requiring 394 participants per group. Assuming approximately 24% attrition at each follow-up assessment, corresponding to an anticipated 12-month follow-up rate of approximately 44% (corresponding to 56% cumulative attrition), a target sample of approximately 1800 participants was planned. No interim analyses were conducted. See the eMethods in Supplement 2 for further details.

### Statistical Analysis

Primary and secondary outcomes were analyzed according to the intention-to-treat principle applying hierarchical linear models (HLMs) (level 1, time; level 2, participants; and level 3, classes), using the R package lme4, version 1.1-37 (R Project for Statistical Computing). Constrained longitudinal data analyses were performed using HLMs including time and group × time interaction as fixed effects. Constrained longitudinal data analysis constrains pretest mean values to be equal between groups. Time was treated as a categorical variable, with baseline as the reference category and separate indicators for the 1-month, 4-month, and 12-month follow-up assessments, accounting for discontinuous change over time. HLMs included random intercepts at levels 2 and 3. Missing data were handled using multiple imputation (stratified by arm). All participants were included after imputation. Prespecified subgroup analyses compared universal and indicated prevention samples. Responder analyses and analyses of harmful effects for the primary outcome were conducted using the Reliable Change Index.^47^ Sensitivity analyses for the primary outcome included completer (ie, provision of complete datasets across all time points) and per-protocol (ie, attendance of all 4 sessions and provision of complete datasets) samples. A detailed description of the statistical models, imputation procedures, and sensitivity analyses is provided in the eMethods in Supplement 2; the statistical analysis code and the complete HLM output are available at PsychArchives.^48^ Statistical significance was defined as a 2-sided *P* < .05.

## Results

### Participant Flow, Sample Characteristics, and Intervention Dose

**Figure 1.**
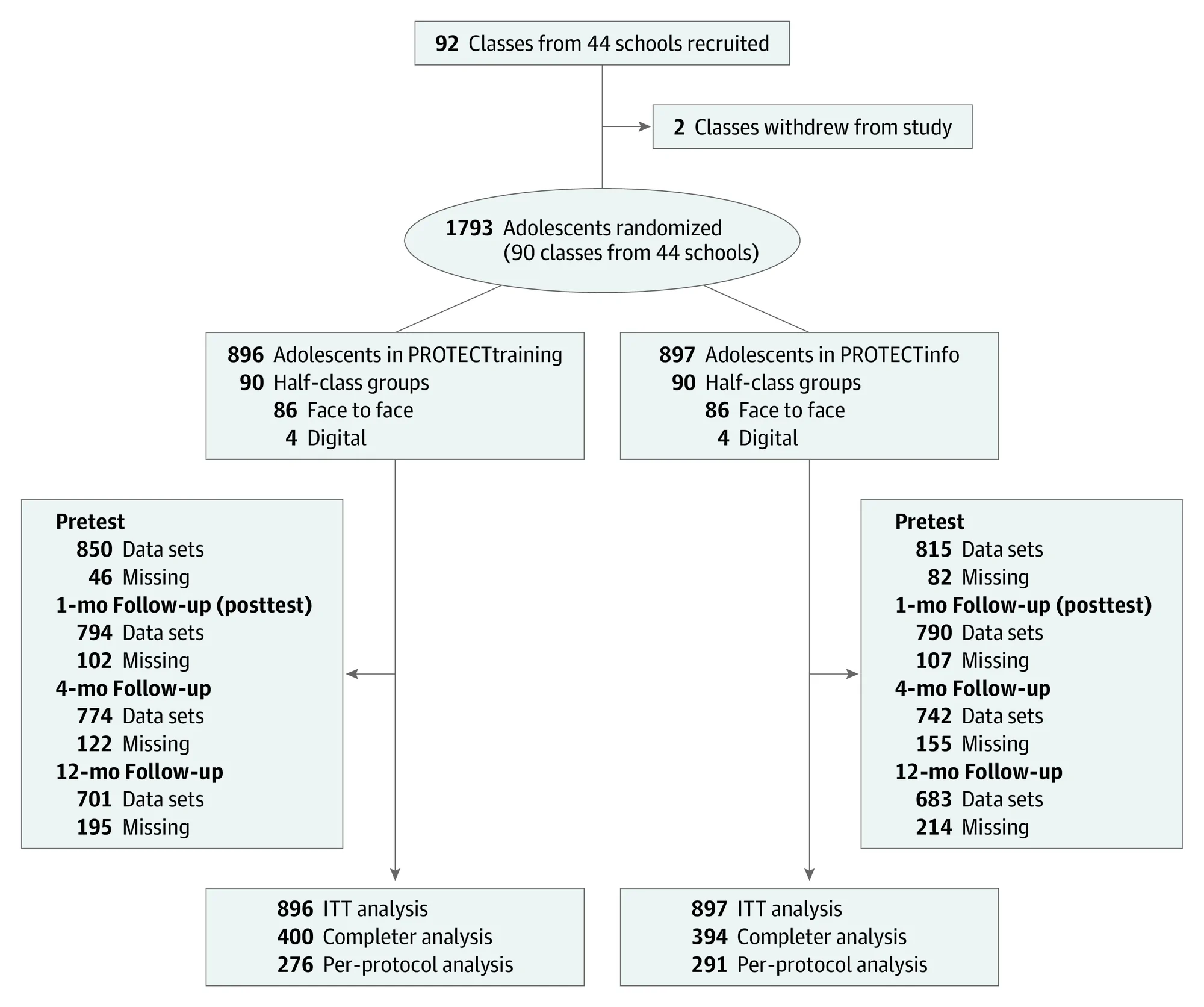
Flow Diagram of Participants Through the PROTECTconfirm Trial Completers were defined as participants who provided complete datasets at all time points. Per-protocol participants were defined as participants who attended all 4 sessions and provided complete datasets at all time points. ITT indicates intention to treat; PROTECT, Professioneller Umgang mit technischen Medien [Professional Use of Technical Media].

Of 2198 students from 92 classes across 44 secondary schools who received either PROTECTinfo or PROTECTtraining in 184 half-class groups, 1793 (81.6%) from 90 classes participated in the study and were individually randomized to the PROTECTtraining condition (n = 896; mean [SD] age, 13.1 [1.5] years; 390 female [43.9%] and 498 male [56.1%] participants; 439 academic track participants [49.7%]) or the PROTECTinfo condition (n = 897; mean [SD] age, 13.1 [1.5] years; 399 female [45.2%] and 484 male [54.8%] participants; 466 academic track participants [52.9%]) (Figure 1 and Table 1). Two classes (4 half-class groups) received the interventions but did not participate in data collection for organizational reasons. There were no further losses after randomization.

**Table 1.** Sample Characteristics at Baseline (Observed Data)^a^

Characteristic	Total sample (N = 1793)	PROTECTtraining (n = 896)	PROTECTinfo (n = 897)
Sociodemographic characteristics			
Age, mean (SD), y	13.1 (1.5)	13.1 (1.5)	13.1 (1.5)
Sex, No. (%)			
Female	789 (44.6)	390 (43.9)	399 (45.2)
Male	982 (55.4)	498 (56.1)	484 (54.8)
Expected degree, No. (%)			
University entrance qualification	905 (51.3)	439 (49.7)	466 (52.9)
Secondary school certificate	831 (47.1)	430 (48.6)	401 (45.5)
Special needs education	5 (0.3)	4 (0.5)	1 (0.1)
No degree	24 (1.4)	11 (1.2)	13 (1.5)
Gaming disorder and internet use disorder symptoms			
Gaming disorder and unspecified internet use disorders, modified CSAS score, mean (SD)	9.33 (8.19)	9.39 (8.12)	9.27 (8.27)
Compulsive internet use, CIUS score, mean (SD)	6.62 (4.16)	6.68 (4.17)	6.56 (4.14)
Gaming attitudes, preoccupation, GAT score, mean (SD)	27.45 (13.32)	27.46 (13.31)	27.43 (13.34)
Gaming attitudes, trivializing, GAT score, mean (SD)	36.38 (14.27)	35.74 (14.40)	37.02 (14.11)
Gaming disorder symptoms, GDT score, mean (SD)	6.61 (2.71)	6.56 (2.57)	6.67 (2.84)
Internet gaming disorder symptoms, IGD-9 score, mean (SD)	1.80 (1.78)	1.85 (1.82)	1.76 (1.74)
Social media disorder symptoms, SMDS score, mean (SD)	1.53 (1.85)	1.56 (1.86)	1.49 (1.84)
Daily time spent gaming, mean (SD), h	2.13 (1.91)	2.10 (1.86)	2.16 (1.95)
Daily time spent on social media, mean (SD), h	2.61 (2.28)	2.68 (2.30)	2.55 (2.25)
Psychopathologic symptoms (transdiagnostic)			
Internalizing psychopathology, SDQ score, mean (SD)	6.09 (3.66)	6.11 (3.60)	6.06 (3.72)
Externalizing psychopathology, SDQ score, mean (SD)	5.82 (3.40)	5.84 (3.43)	5.80 (3.36)
HrQoL, KIDSCREEN-27 score, mean (SD)	106.97 (14.48)	106.71 (14.79)	107.24 (14.77)
Well-being, WHO-5 score, mean (SD)	15.23 (5.56)	15.16 (5.65)	15.31 (5.48)
Psychopathologic symptoms (disorder specific)			
Depressive symptoms, DesTeen score, mean (SD)	10.70 (7.03)	10.48 (6.92)	10.92 (7.14)
Social anxiety symptoms, SIAS score, mean (SD)	22.69 (13.45)	22.56 (13.40)	22.84 (13.50)
Performance anxiety symptoms, FSSC-R score, mean (SD)	6.90 (4.12)	6.71 (4.19)	7.09 (4.04)
ADHD symptoms, Conners-3 score, mean (SD)	7.96 (4.99)	7.98 (5.07)	7.93 (4.91)
Mechanisms of change			
Procrastination, PFS-4 score, mean (SD)	8.86 (3.76)	8.73 (3.70)	8.98 (3.82)
Emotion dysregulation, DERS-SF score, mean (SD)	38.11 (12.94)	37.74 (12.87)	38.49 (13.01)

Abbreviations: ADHD, attention-deficit/hyperactivity disorder; CIUS, Compulsive Internet Use Scale; Conners-3, Conners 3rd Edition; CSAS, modified version of the Video Game Dependency Scale [Computerspielabhängigkeitsskala]; DERS-SF, 18-item version of the Difficulties in Emotion Regulation Scale; DesTeen, Depression Screener for Teenagers; FSSC-R, Fear Survey Schedule for Children–Revised; GAT, Gaming Attitude Test; GDT, Gaming Disorder Test; IGD-9, Internet Gaming Disorder Scale; KIDSCREEN-27, 27-item version of the KIDSCREEN questionnaire; PFS-4, 4-item version of the Procrastination Questionnaire for Students; PROTECT, Professioneller Umgang mit technischen Medien [Professional Use of Technical Media]; SDQ, Strength and Difficulties Questionnaire; SIAS, Social Interaction Anxiety Scale; SMDS, Social Media Disorder Scale; WHO-5, World Health Organization Well-Being Index.

^a^ German school grading system reaches from 1 (very good) to 6 (insufficient). Daily time spent gaming and on social media was capped at 12 hours.

The interventions were evaluated within 180 groups (6-16 students per group), with a mean of 10 study participants per group (range, 2-16 study participants per group). The mean (SD) number of sessions attended was 3.5 (0.8) of 4 sessions in PROTECTtraining and 3.4 (0.8) in PROTECTinfo.

Baseline data were available for 94.9% of PROTECTtraining participants (850 of 896) and 90.9% of PROTECTinfo participants (815 of 897). Attrition at 12 months was 21.8% (195 of 896) in PROTECTtraining and 23.9% (214 of 897) in PROTECTinfo. High-risk status, based on the modified CSAS, was identified in 11.4% of all participants (n = 205): 11.6% of PROTECTtraining participants (104 of 896) and 11.3% of PROTECTinfo participants (101 of 897).

### Primary Outcome

**Figure 2.**
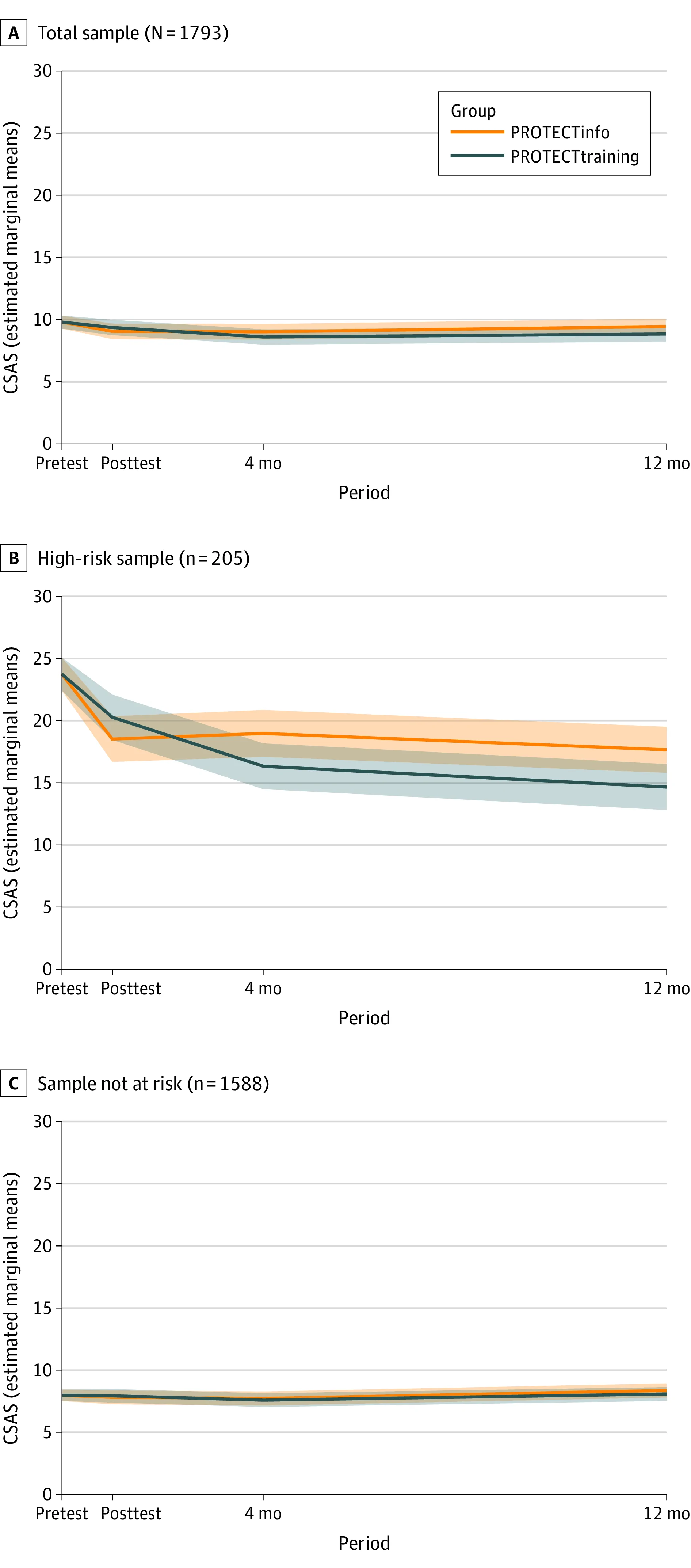
Line Graphs Showing Gaming Disorder and Unspecified Internet Use Disorder Symptom Changes Over 12 Months Shaded areas indicate 95% CIs. CSAS indicates the modified Video Game Dependency Scale (Computerspielabhängigkeitsskala); PROTECT, Professioneller Umgang mit technischen Medien [Professional Use of Technical Media].

In the intention-to-treat analysis, severity of gaming disorder or unspecified internet use disorder symptoms did not differ significantly between groups (PROTECTtraining: mean [SD] CSAS score, 8.8 [8.3] vs PROTECTinfo: mean [SD] CSAS score, 9.4 [8.4]; within-group Cohen *d* = −0.11 [95% CI, −0.18 to −0.05] vs −0.04 [95% CI, −0.11 to 0.02]; group × time at 12 months: *b* = −0.60; *P* = .09) (Table 2; Figure 2A). Among adolescents classified as high risk (n = 205), both programs showed strong within-group improvement, but the CBT-based PROTECTtraining yielded a significantly greater reduction (within-group Cohen *d* = −0.91 [95% CI, −1.10 to −0.72] vs −0.61 [95% CI, −0.80 to −0.42]; group × time at 12 months: *b* = −3.00; *P* = .02; mean [SD] CSAS score: PROTECTtraining, 14.7 [9.7] and PROTECTinfo, 17.7 [10.3]; between-group Cohen *d* = −0.30 [95% CI, −0.55 to −0.05]) (Table 2; Figure 2B). In the subgroup not at risk (n = 1588), no significant group × time effect at 12 months emerged (*b* = −0.29; *P* = .41) (Table 2; Figure 2C).

**Table 2.** Primary Outcome: Estimated Marginal Mean Values and Effect Sizes After Multiple Imputation (Intention-to-Treat Analyses)^a^

Sample	Mean (SD) Modified Video Game Dependency Scale score				Within-group Cohen *d* (95% CI) (pretest vs 12 mo)
	Pretest	Posttest	At 4 mo	At 12 mo	
**Total sample (N = 1793)^b^**					
PROTECTtraining	9.8 (8.4)	9.4 (8.7)	8.6 (8.4)	8.8 (8.3)	−0.11 (−0.18 to −0.05)
PROTECTinfo	9.8 (8.6)	9.1 (8.3)	9.0 (9.0)	9.4 (8.4)	−0.04 (−0.11 to 0.02)
Between-group Cohen *d* (95% CI)	NA	0.04 (−0.04 to 0.12)	−0.05 (−0.13 to 0.03)	−0.07 (−0.16 to 0.01)	NA
*P* value	NA	.38	.24	.09	NA
**High-risk sample (n = 205)^c^**					
PROTECTtraining	23.7 (7.4)	20.3 (10.0)	16.3 (9.6)	14.7 (9.7)	−0.91 (−1.10 to −0.72)
PROTECTinfo	23.7 (8.1)	18.5 (10.0)	19.0 (11.3)	17.7 (10.3)	−0.61 (−0.80 to −0.42)
Between-group Cohen *d* (95% CI)	NA	0.18 (−0.07 to 0.42)	−0.25 (−0.49 to −0.01)	−0.30 (−0.55 to −0.05)	NA
*P* value	NA	.16	.04	.02	NA
**Sample not at risk (n = 1588)^b^**					
PROTECTtraining	8.0 (6.7)	7.9 (7.5)	7.6 (7.7)	8.1 (7.8)	0.01 (−0.05 to 0.08)
PROTECTinfo	8.0 (6.7)	7.8 (7.2)	7.7 (7.8)	8.4 (7.4)	0.05 (−0.02 to 0.12)
Between-group Cohen *d* (95% CI)	NA	0.02 (−0.07 to 0.11)	−0.02 (−0.10 to 0.07)	−0.04 (−0.13 to 0.05)	NA
*P* value	NA	.72	.71	.41	NA

Abbreviations: NA, not applicable; PROTECT, Professioneller Umgang mit technischen Medien [Professional Use of Technical Media].

^a^ Time was treated as a factor (0, 1, 4, and 12 months). Primary outcome is the modified version of the Video Game Dependency Scale score at 12 months to capture gaming disorder and unspecified internet use disorder.

^b^ Three-level model (observations in participants in classes).

^c^ Two-level model (observations in participants). Estimated marginal mean values were derived from hierarchical linear models including time and the group × time interaction as fixed effects.

### Sensitivity Analysis and Secondary Outcomes

These findings remained stable across sensitivity analyses, including completer analyses (eTable 2 in Supplement 2) and per-protocol analyses (eTable 3 in Supplement 2), all confirming the direction and magnitude of the primary intention-to-treat effect. No significant between-group differences were observed for any of the secondary outcomes (eTable 4 in Supplement 2). Despite the absence of significant interaction effects in HLM models, adolescents at high risk who received PROTECTtraining vs PROTECTinfo showed reduced SDQ internalizing symptoms scores (PROTECTtraining pretest vs 12 months: within-group Cohen *d* = −0.20 [95% CI, −0.40 to 0.00]; PROTECTinfo pretest vs 12 months: within-group Cohen *d* = 0.05 [95% CI, −0.17 to 0.27]).

### Responder Analyses

Reliable improvement occurred in 12.7% of PROTECTtraining participants (71 of 557) and 9.9% of PROTECTinfo participants (53 of 538) (eTable 5 in Supplement 2). In the subgroup of adolescents at high risk, reliable improvement rates were 46.4% among PROTECTtraining participants (26 of 56) vs 38.6% PROTECTinfo participants (22 of 57). The frequency of reliable improvement did not differ significantly between groups (all *P* > .05).

### Harms and Concomitant Care

Reliable deterioration occurred in 7.9% of PROTECTtraining participants (44 of 557) and 9.1% of PROTECTinfo participants (49 of 538) (eTable 6 in Supplement 2). The lowest rate of deterioration was observed in the subgroup of adolescents at high risk receiving the CBT intervention (3.6% [2 of 56]). Concomitant care was not assessed. The frequency of reliable deterioration did not differ significantly between groups.

### Implementation Fidelity and Satisfaction

Intervention fidelity was high in both interventions (eTable 7 in Supplement 2). Satisfaction was higher for PROTECTinfo than PROTECTtraining (mean [SD], 7.41 [2.18] vs 6.60 [2.54]; *P* < .001).

## Discussion

This multicenter randomized clinical trial demonstrated that the CBT-based PROTECTtraining program reduced gaming disorder symptoms and unspecified internet use disorder symptoms over 12 months under routine school conditions by trained local facilitators. Moreover, PROTECTtraining was superior to a structurally parallel, ML-based active control intervention (PROTECTinfo) among adolescents at high risk, supporting the relevance of indicated prevention.

### Effectiveness Under Naturalistic Conditions

These findings extend previous efficacy evidence to large-scale routine school implementation as a translational step. Whereas the initial trial^18^ demonstrated efficacy against an assessment-only control condition under delivery by trained psychologists, the present study evaluated implementation under routine school conditions. Together with high fidelity ratings, this finding suggests that manualized CBT-based prevention can be delivered effectively by trained local professionals.

### CBT-Based vs ML-Based Content of the Intervention

Both interventions were associated with symptom reductions. However, PROTECTtraining (CBT based) yielded significantly stronger effects in HLM models on gaming disorder symptoms and unspecified internet use disorder symptoms in the group of adolescents at high risk. The effects observed in PROTECTinfo were approximately two-thirds the magnitude of those achieved with PROTECTtraining (small between-group effect size). Although PROTECTinfo received higher satisfaction ratings, PROTECTtraining produced significantly larger effects. These findings suggest that school prevention programs should prioritize evidence-based effectiveness over acceptability alone.

### Universal vs Indicated Prevention

Clinically relevant symptoms are relatively infrequent in the general population, typically develop over extended periods, and may also be episodic. Adolescents at high risk, comprising 11.4% of the total sample, benefited substantially from both interventions, particularly from CBT, whereas no effects were observed in the much larger subsample of adolescents not at risk. Indicated prevention approaches are therefore both more effective and more resource efficient than purely universal strategies, and prior research has demonstrated the feasibility of risk screening and indicated prevention in school settings.^18^ Although universal school-based delivery is logistically appealing, the nonsignificant effects observed in the general population suggest that stepped prevention (universal screening followed by indicated intervention) may be more effective and resource efficient.

### Specificity of Intervention Effects

Intervention effects were largely specific to the intervention’s primary target (ie, symptoms of gaming disorder and unspecified internet use disorder). Effects on SDQ internalizing symptoms were small, observed only in the CBT condition, and did not result in significant between-group differences. The use of a structurally parallel active control provides a more stringent test of content specificity than the prior assessment-only design.

### Balancing Benefits and Harms

Both interventions were well tolerated, with reliable improvements occurring 4 to 5 times more frequently than reliable deteriorations over 12 months. The lowest rate of deterioration was observed in the subgroup of adolescents at high risk receiving the CBT intervention (3.6%), which simultaneously showed the highest proportion of reliable improvement (46.4%).

### Strengths and Limitations

Key strengths of this study include its large and representative school-based sample, individual randomization within classes, implementation by trained local professionals, and rigorous supervision and fidelity monitoring, ensuring protocol adherence. The design’s ecological validity enhances generalizability and provides a realistic benchmark for dissemination in educational systems.

Several limitations merit consideration. First, structured clinical interviews were not feasible in this large school-based sample, precluding clinical ratings of gaming disorder and internet use disorder. Second, although fidelity ratings indicated high adherence, variation in facilitator competence and background may have influenced effect sizes; these differences warrant further investigation. Third, the 12-month follow-up provides limited insight into long-term prevention of disorder onset. Fourth, intervention effects were evident only on the modified CSAS, whereas the other scales (short CIUS, IGD-9, and GDT) showed no significant group differences. This may be due to the CSAS’s greater length (18 items vs ≤9 items), its item response format (5-point Likert scale vs dichotomous format in IGD-9), its adolescent adaptation, and its coverage of both gaming and other internet behaviors. Fifth, the primary outcome was based on a *DSM-5* conceptualization to replicate the original trial conducted before the publication of *ICD-11*. Sixth, the study was conducted in one German federal state, which may limit generalizability.

## Conclusions

In this randomized clinical trial of 1793 adolescents, CBT-based prevention of gaming disorder and unspecified internet use disorder was more effective than ML-based prevention among adolescents at high risk, but not among the total sample. These findings support the superiority of CBT-based approaches over ML-based interventions and support their use primarily within indicated prevention (high-risk group), but not universal prevention (total sample).

These findings indicate that person-delivered CBT-based prevention of gaming disorder and unspecified internet use disorder can be effectively implemented in school settings, particularly for adolescents at high risk. Standardized facilitator training, live supervision, and systematic fidelity monitoring appear to be a central determinant of successful and scalable implementation. In further analyses, we will examine the role of facilitators’ professional backgrounds (eg, teachers, social workers, psychologists) and treatment fidelity in explaining variability in intervention effects. Understanding how these factors interact will be essential for optimizing dissemination strategies. In addition, planned health economic analyses will quantify cost-effectiveness differences between the respective prevention approaches. App-delivered (PROTECTapp^49,50^) and chatbot-supported (PROTECTai; German Clinical Trials Register [DRKS] trial registration DRKS00036567) adaptations are currently under evaluation and may offer scalable alternatives if effective. Future research should clarify which intervention techniques (eg, inhibitory control vs emotion regulation) drive preventive effects, as examined in the European BootStRaP (Boosting Societal Adaptation and Mental Health in a Rapidly Digitalizing, Post-Pandemic Europe) trial.^51^

## Notes

Supplement 1.
Trial Protocol and Statistical Analysis Plan

Supplement 2.
eMethods.
**eTable 1. **Measures and Assessment Time Points **eTable 2. **Completer Analysis. Estimated Marginal Means, Observed *SD*s, and Effect Sizes of the Primary Outcome **eTable 3. **Per-Protocol Analysis. Estimated Marginal Means, Observed *SD*s, and Effect Sizes of the Primary Outcome **eTable 4. **Secondary Outcomes. Estimated Marginal Means, Observed *SD*s, and Effect Sizes of Secondary Outcomes After Multiple Imputation (Intention-to-Treat Analyses) **eTable 5.** Responder Analysis. Frequencies of Reliable Improvement From Pretest to 12-Months Follow-Up on Primary (Observed Data) **eTable 6. **Negative Effects. Frequencies of Reliable Deterioration From Pretest to 12-Months Follow-Up on Primary Outcome (Observed Data) **eTable 7. **Adherence. Descriptive Statistics of Adherence Ratings
eReferences.

Supplement 3.
Data Sharing Statement
