# Supplementary material for: Cognitive Behavioral Therapy vs Media Literacy for Prevention of Gaming Disorder and Unspecified Internet Use Disorder: A Randomized Clinical Trial
Source: JAMA Netw Open. 2026 Sep 4;9(9):e2631942. doi: 10.1001/jamanetworkopen.2026.31942 (PMC13545672; doi:10.1001/jamanetworkopen.2026.31942)
Supplement: Supplementary file 1 [file jamanetwopen-e2631942-s001.pdf]

Study protocol

Effectiveness of the school-based primary prevention program "PROTECT" for the prevention of gaming disorder and problematic internet use under real-world conditions in 15 regional centers in Baden-Württemberg

Acronym: PROTECTconfirm

Principal Investigator

Prof. Dr. Katajun Lindenberg
Kinder- und Jugendlichenpsychotherapie
Goethe-Universität Frankfurt | Campus Bockenheim
Carl-Bosch-Haus | Varrentrappstraße 40-42
60486 Frankfurt am Main

Additional Collaborators

Prof. Dr. habil. Lutz Wartberg
Professur für Gesundheitspsychologie

MSH Medical School Hamburg
Am Kaiserkai 1
20457 Hamburg

M.Sc. Psych. Sarah Hofmann
M.Sc. Psych. Sonja Kewitz
Dipl.-Psych. Katharina Leo

Goethe-Universität Frankfurt | Campus Bockenheim
Carl-Bosch-Haus | Varrentrappstraße 40-42
60486 Frankfurt am Main

Table of Content

1 General Information 3
1.1 Applicant / Project Lead 3
1.2 Topic 3
1.3 Project Title 3
1.4 Discipline and Research Focus 3
1.5 Funding Period 3
1.6 Supporting Institutions, Companies, and Organizations 3
1.7 Summary 3
2 State of the Art and Previous Work 4
2.1 State of the Art 4
2.2 Description of Previous Work 6
3 Objectives and Work Program 7
3.1 Objectives 7
3.1.1 Theoretical Rationale of the Project 7
3.1.2 Objectives and Hypotheses / Research Questions 8
3.1.3 Primary Outcome 9
3.1.4 Secondary Outcomes 9
3.1.5 Rationale for the Study Design Based on the Research Question 9
3.2 Work Program 10
3.2.1 Implementation of the study 10
3.2.2 Sample size 12
3.2.3 Randomization 12
3.2.4 Inclusion and Exclusion Criteria 12
3.2.5 Measures and Control Variables 12
3.2.6 Study design 15
3.2.7 Statistical Analyses / Data Analysis 15
3.2.8 Work Plan and Timeline With Milestones 16
3.3 Studies Involving Human Participants 16
3.3.1 Safety Measures for Study Participants 16
3.3.2 Criteria for Study Discontinuation 17
3.3.3 Ethical and Legal Considerations 17
3.4 Data Protection 17
References 19

1 General Information

1.1 Applicant / Project Lead

Prof. Dr. Katajun Lindenberg, Abteilung Kinder- und Jugendlichenpsychotherapie
Fachbereich Psychologie und Sportwissenschaften, Institut für Psychologie

1.2 Topic

Confirmatory evaluation of the effectiveness of the primary preventive intervention PROTECT for the prevention of gaming disorder and internet addiction under real-world conditions

1.3 Project Title

PROTECTconfirm (Acronym für „Professioneller Umgang mit technischen Medien“)

1.4 Discipline and Research Focus

Discipline: Clinical psychology, child and adolescent psychotherapy
Research focus: Quantitative-empirical psychological prevention research in schools

1.5 Funding Period

Start of funding: July 1, 2020
Duration: until August 31, 2024

1.6 Supporting Institutions, Companies, and Organizations

The project presented here is funded by the German Federal Ministry of Education and Research (BMBF). Statistical analyses are supported by the Medical School Hamburg (MSH).

The project is conducted in close cooperation with the PROTECTdissemination project, which is funded by the Ministry of Social Affairs and Integration of Baden-Württemberg. There is close collaboration with the municipal addiction commissioners in Baden-Württemberg. Additional cooperation structures exist with the Karlsruhe Regional Council, the delegated prevention officers, the addiction support network of the Rhine-Neckar district, and the municipal addiction commissioners of the Rhine-Neckar district, Mannheim, and Heidelberg.

1.7 Summary

High prevalence rates of pathological internet use and the associated psychosocial impairments highlight the need for effective and cost-efficient measures for prevention and early intervention. With the inclusion of (Internet) Gaming Disorder in the current classification systems of mental disorders (DSM-5; American Psychiatric Association, 2013, and ICD-11; World Health Organization, 2018), there has been a targeted call for the development of prevention and early intervention measures, as prevention and treatment research in this young disorder category—particularly in

childhood and adolescence—is still largely pioneering work. Our research group has focused specifically on this research area.

The prevention program “PROTECT” for the prevention of gaming disorder and internet addiction currently has the strongest evidence base internationally. Sustainable reductions in core symptoms have been demonstrated in several studies. Beyond statistical and clinical effectiveness, acceptance of the program in schools is high.

The aim of the present project, PROTECTconfirm, is the confirmatory evaluation of the effectiveness of the primary preventive intervention PROTECT for the prevention of gaming disorder and internet addiction under real-world conditions. The project builds on the PROTECTdissemination project funded by the Ministry of Social Affairs and Integration of Baden-Württemberg, which aims to establish a dissemination structure that enables PROTECT to be implemented broadly and sustainably in schools throughout Baden-Württemberg.

In the controlled, multicenter, confirmatory study presented here, the prevention infrastructure established within the PROTECTdissemination project will be used to collect new data using the study design of the original PROTECT trial. The effectiveness of PROTECT prevention will thus be examined under real-world conditions across different contexts. The large sample size (N = 1,800) provides a robust data basis for examining interactions related to psychological well-being, quality of life, and comorbid symptoms. In addition to validating previous findings, further analyses of specific mechanisms of action (component analyses), differential effects (e.g., by usage intensity and application form), and health economic questions (cost-utility analyses) will be conducted. These analyses are particularly relevant for long-term cost coverage by health care providers.

The study targets 90 school classes from 15 regional centers in Baden-Württemberg and is planned for a duration of three years.

2 State of the Art and Previous Work

2.1 State of the Art

With the inclusion of problematic computer gaming, referred to as (Internet) Gaming Disorder (IGD or GD), in the current classification systems of mental disorders (DSM-5 in 2013; American Psychiatric Association; and ICD-11 in 2018; World Health Organization), the pathological nature of this relatively young phenomenon has been formally recognized and there has been a targeted call for research on prevention and early intervention measures. Gaming Disorder (according to ICD-11) is characterized by the following core features: (1) impaired control over gaming (onset, frequency, intensity, duration, context); (2) increasing priority given to gaming over other life interests and daily activities; and (3) continuation or escalation of gaming despite negative consequences.

In addition to gaming disorder, other forms of excessive internet use (e.g., online communication, online shopping, or online pornography) are associated with comparable psychosocial impairments and an increased risk of addiction. Although these behaviors have not yet been included as independent diagnostic entities due to insufficient empirical evidence, they are taken into account in existing primary prevention approaches. Both diagnostic systems explicitly call for further research on these application forms.

From an epidemiological perspective, the prevention of gaming disorder and internet addiction is of high relevance. In Germany, prevalence estimates of gaming disorder in adolescence range between 1.2% and 5.7%. The prevalence of internet addiction, independent of the specific application, is estimated to range between 3.2% and 6.1% among German adolescents.

The goals of prevention include the modification of risk factors, the prevention of core symptoms, and the avoidance of negative secondary consequences. The significance and severity of gaming disorder and internet addiction are particularly evident in the high rates of comorbid mental disorders reported in the literature. These include depression, anxiety disorders, obsessive-compulsive disorders, conduct disorders, attention-deficit/hyperactivity disorder, and substance-related disorders. Although the underlying causal mechanisms are not yet fully understood, reciprocal interactions between these disorders appear most likely.

Individuals affected by gaming disorder and internet addiction rarely seek treatment on their own initiative, which complicates timely intervention. Low-threshold preventive programs, particularly in school settings, enable early access to support services for at-risk users and may prevent the manifestation of the disorder. An additional advantage of school-based prevention is the ability to reach entire cohorts due to compulsory school attendance, as well as the potential involvement of parents.

From a clinical perspective, preventing or delaying the onset of gaming disorder and internet addiction and associated comorbidities through primary preventive measures is highly relevant, as this improves quality of life and increases the number of healthy life years (e.g., measured as quality-adjusted life years, QALYs) in the population.

Beyond its clinical relevance, prevention is also of major importance from a health policy and economic perspective. Mental and behavioral disorders represent the second highest cost category in the German health care system, ranking just behind cardiovascular diseases.

International review studies report between eight and twenty prevention programs worldwide. Only a small proportion of these programs have examined reductions in core symptoms as a primary endpoint, and the methodological quality of many studies has been critically appraised. The PROTECT program developed by our research group—a cognitive-behavioral primary prevention program implemented in school settings—has an internationally leading position and currently represents the strongest evidence base. The PROTECT research program was therefore awarded the Hermann Hemminghaus Prize of the German Society for Child and Adolescent Psychiatry and Psychotherapy in 2019.

2.2 Description of Previous Work

Our research group (principal investigator: Prof. Dr. Katajun Lindenberg) focuses on the prevention of mental disorders in childhood and adolescence. Cooperative structures exist, among others, with the Ministry of Social Affairs and Integration of Baden-Württemberg, the Karlsruhe Regional Council and its prevention officers, the addiction support network of the Rhine-Neckar district, and the municipal addiction commissioners of the Rhine-Neckar district, Mannheim, and Heidelberg.

In 2013, our research group developed a cognitive-behavioral intervention (PROTECT – Professional Use of Technical Media), which has been evaluated as a prevention program in several studies. The PROTECT program includes psychoeducation, behavioral modification (development of alternative activities and problem-solving training), improvement of emotion regulation, and cognitive restructuring. The prevention program consists of four modules of 90 minutes each and was delivered by study therapists.

In a school-based setting, a randomized controlled efficacy trial with a 12-month follow-up was conducted with N = 480 adolescents at elevated risk (study period 05/2015–09/2018; registration: ClinicalTrials.gov: NCT02907658). Symptoms of gaming disorder and internet addiction were assessed using the Compulsive Internet Use Scale and the Computer Gaming Addiction Scale. Data were analyzed using multilevel modeling. Results showed a significant reduction in core symptoms of gaming disorder and internet addiction after 12 months, with a significant time-by-group interaction.

In a further study (study period 04/2016–03/2019; registration: ClinicalTrials.gov: NCT03582839), the effectiveness of PROTECT as an early intervention measure (PROTECT+) was examined in N = 54 adolescent patients in an outpatient group therapy setting. Here as well, a mid-term reduction in core symptoms was observed, while analyses of long-term effects are still pending. A third study (PROTECT 3–5) investigated the effectiveness of an adapted version of the program for a younger age group (students in grades 3 to 5).

Building on the scientific success of the original PROTECT trial, a dissemination structure (PROTECTdissemination) has been established since November 2019 through funding from the Ministry of Social Affairs and Integration of Baden-Württemberg. This structure enables the large-scale and sustainable implementation of PROTECT throughout Baden-Württemberg. Within the PROTECTdissemination project, 15 regional centers consisting of municipal addiction commissioners and staff from counseling services with a prevention mandate in schools have been established. Over the three-year funding period, a total of 90 trainers are trained and certified, who independently deliver PROTECT prevention in 90 school classes. This infrastructure provides the basis for a multicenter study including 1,800 students from 90 classes across 15 regional centers.

3 Objectives and Work Program

3.1 Objectives

3.1.1 Theoretical Rationale of the Project

The funding guideline for research aimed at strengthening evidence-based practice and knowledge transfer in prevention research seeks to promote and maintain population health through the targeted investigation of primary preventive interventions. In particular, the investigation of long-term effects of already implemented measures is a key priority, as such evidence is essential for successful large-scale transfer. In line with this objective, the PROTECT program for the prevention of gaming disorder and internet addiction—which has previously been successfully evaluated under research conditions—is now to be evaluated under real-world conditions in schools, that is, delivered by trained and certified multipliers.

The starting point for the present project is an undersupply of preventive measures addressing gaming disorder and internet addiction among young people, despite high prevalence rates and substantial associated psychosocial impairments. High treatment costs, reduced educational opportunities, and increased unemployment rates further underscore the health policy and educational relevance of prevention. With the inclusion of problematic gaming behavior ((Internet) Gaming Disorder) in the current classification systems of mental disorders (DSM-5 in 2013; ICD-11 in 2018), there has been an explicit call for the development of prevention and early intervention strategies, as existing approaches—particularly for children and adolescents—are largely not evidence-based.

The confirmatory study presented here is therefore of high research policy relevance, as it provides a critical empirical basis for future reimbursement decisions by health care payers. Gaming disorder and internet addiction typically emerge during adolescence, with prevalence increasing into young adulthood. While prevalence among 11- to 12-year-olds is approximately 2.8%, it rises to up to 9.1% among 19- to 21-year-olds. Adolescence therefore represents a particularly important window for preventive interventions.

At the same time, adolescents from approximately 12 years of age typically possess sufficient cognitive capacities to apply the cognitive techniques included in the PROTECT prevention program and to complete the study questionnaires. In addition, school organizational structures in grades 7 to 10 generally provide time resources for preventive interventions, making this age range highly compatible with everyday school practice.

PROTECTconfirm aims to examine the effectiveness and long-term effects of the preventive intervention under real-world conditions in a multicenter study. In contrast to the previous efficacy trial conducted under controlled research conditions, the intervention will be delivered as part of routine school-based prevention services across diverse settings, including underserved regions, different school types, and varying age groups, by trained professionals such as school social workers and addiction counselors.

This design ensures high external validity through implementation under real-world conditions (effectiveness study). High internal validity is maintained through standardized, manual-based delivery under supervision, a controlled longitudinal study design with four assessment points, standardized diagnostics, and the use of hierarchical linear modeling and structural equation modeling to control for potential confounding variables.

3.1.2 Objectives and Hypotheses / Research Questions

The overarching scientific objective of the present project is to establish an evidence base for the PROTECT primary prevention program under real-world conditions and across different contexts. The results provide a crucial foundation for the health care system, as evidence of effectiveness is essential for reimbursement by relevant payers (e.g., statutory health insurance). Accordingly, additional health economic analyses examine the costs and benefits of prevention, including quality-adjusted life years (QALYs) and avoided treatment costs, to estimate potential cost savings for society.

Under controlled research conditions (efficacy), the effectiveness of PROTECT prevention has already been demonstrated in a randomized controlled trial and additional studies. These studies prioritized internal validity. In contrast, the aim of the present study is to enhance external validity by examining effectiveness under real-world conditions, without preselection of participants, thereby accurately reflecting everyday practice.

The study generates a robust data base for subgroup analyses, interaction analyses, and the investigation of mechanisms of action of the preventive intervention through mediator and moderator analyses.

3.1.2.1. Main Hypotheses / Research Questions

Hypothesis 1a: The PROTECTtraining prevention program leads to a significant reduction in symptoms of gaming disorder and internet addiction under real-world conditions.

Adolescents at elevated risk (top 20%) benefit more from the intervention than adolescents without elevated risk.

Hypothesis 1b: The media literacy program PROTECTinfo also leads to a reduction in symptoms or prevents an increase in symptoms of gaming disorder and internet addiction.

Hypothesis 1c: Adolescents at elevated risk benefit more from cognitive-behavioral prevention (PROTECTtraining) than from media literacy prevention.

Hypothesis 1d: The reduction in symptoms of gaming disorder and internet addiction is significantly greater for students who complete PROTECTtraining than for students who complete the active control program PROTECTinfo.

Research Question 1: Does the PROTECTinfo program lead to a significant reduction in symptoms of gaming disorder and internet addiction?

3.1.2.2. Secondary Hypotheses / Research Questions

Hypothesis 2: Students and multipliers evaluate the PROTECT prevention program positively under real-world conditions, with ratings above the scale midpoint.

Research Question 2a: What interaction effects and mechanisms of action characterize the PROTECTtraining prevention program, particularly in relation to psychological well-being, quality of life, and comorbid symptoms?

Research Question 2b: What interaction effects and mechanisms of action characterize the PROTECTinfo prevention program, particularly in relation to psychological well-being, quality of life, and comorbid symptoms?

Research Question 3: Do findings differ by region, school type, and/or sociodemographic characteristics?

Research Question 4: What are the results of the health economic analyses, and what potential cost savings for social systems can be identified?

3.1.3 Primary Outcome

- Validation of previous findings under real-world conditions, defined as a reduction in symptoms of gaming disorder and internet addiction.

3.1.4 Secondary Outcomes

- Demonstration of feasibility and acceptance of the PROTECT prevention program under real-world conditions;
- analysis of interaction effects and mechanisms of action (mediator and moderator analyses);
- investigation of differential effectiveness across contexts (regional differences, school types, sociodemographic factors);
- health economic analyses (cost-utility analyses) and estimation of potential cost savings for social systems.

3.1.5 Rationale for the Study Design Based on the Research Question

he present study is a two-arm, controlled, multicenter, prospective long-term study with four measurement points (baseline, 1 month, 4 months, 12 months). The long-term assessment allows for the analysis of differences in trajectories up to 12 months after the start of the intervention. Allocation to the two groups, PROTECTinfo and PROTECTtraining, is carried out by randomization. The active control group allows the effect of the PROTECTtraining prevention program to be tested in reference to the information-based PROTECTinfo program.

A confirmatory study on the effectiveness of real-world measures under everyday conditions, in accordance with the German Federal Ministry of Education and Research, should adhere as closely as possible to the original study, so that the present study design corresponds to that of the original PROTECT study (Lindenberg, Halasy, & Schoenmaekers, 2017). To ensure testing under real-world conditions in the current study, the following deviations from the original study are made: There is no preselection of students. In addition, the intervention is delivered not by study therapists but by trained addiction professionals. Furthermore, instead of a passive control group, an active control group is used that completes the time-equivalent psychoeducational program PROTECTinfo. Finally, secondary outcomes are in part assessed using different measurement instruments. In this way, the constructs can be optimized and assessed efficiently.

3.2 Work Program

The PROTECT prevention program for the prevention of gaming disorder and internet addiction (Lindenberg, Halasy, & Schoenmaekers, 2017) is a cognitive-behavioral intervention aimed at promoting healthy behavioral patterns and ways of thinking. PROTECT is targeted at students aged 12 to 18 years and consists of four 90-minute modules. The prevention program includes two levels of intensity: PROTECTinfo is primarily psychoeducational in nature, whereas PROTECTtraining focuses on the teaching of cognitive techniques, problem-solving skills, and emotion regulation skills. The core objectives are (1) coping with boredom and motivational difficulties, (2) reduction of procrastination and performance anxiety, (3) coping with social insecurity, and (4) promotion of emotion recognition and emotion regulation skills. Detailed information on the contents of PROTECTtraining can be found in the PROTECT manual (Lindenberg & Research Group on Gaming and Internet Addiction, 2018). The contents of PROTECTinfo are available in manualized form as an internal publication of the Department of Child and Adolescent Psychotherapy at Goethe University Frankfurt.

3.2.1 Implementation of the study

The study is conducted in schools located within the catchment areas of the 15 regional centers in Baden-Württemberg. These schools differ with regard to their location (urban and rural areas) as well as school type (academic-track secondary schools, intermediate secondary schools, and lower-track secondary schools). By explicitly including rural and potentially underserved regions, the study aims to reduce social inequalities.

The target group consists of students from grade 7 onwards. These students are recruited from the catchment areas of the 15 regional centers, within which 90 multipliers are trained (approximately six per center) who are responsible for delivering the preventive intervention. The regional centers are coordinated by the responsible municipal addiction commissioners.

Implementation of the prevention program takes place under real-world conditions as part of the regular prevention services offered in schools, such that participation does not result in the loss of regular class time. As a rule, the entire class participates in PROTECT, with the intervention delivered in one of two variants (PROTECTinfo or PROTECTtraining) in small groups (half-class size, at least ten students). The school setting provides optimal access to the target group. Cooperation between schools and the established prevention professionals responsible for implementation is intended to increase schools' willingness to participate. The implementation of the prevention program and the recruitment of participating service providers (multipliers) are secured and are not the subject of the present project.

In total, 1,800 students from 90 classes will receive PROTECT as a primary preventive intervention. Each multiplier delivers both a PROTECTtraining and a PROTECTinfo group, resulting in a total of 900 students participating in PROTECTtraining and 900 students participating in the PROTECTinfo program. To validate previous findings, the optimized assessment schedule of the original study is used (Lindenberg, Halasy, & Schoenmaekers, 2017).

Students are informed in advance about the modules and the subsequent assessments by the multipliers. In addition, parents receive a detailed information letter about the interventions and assessments, as well as a consent form for their children's participation. Data collection within the framework of this study primarily takes place at school.

If required due to the circumstances of the COVID-19 pandemic, surveys may be conducted online via the Unipark platform (www.unipark.com
). All data are collected in pseudonymized form regardless of the mode of assessment, by encrypting them using a code and storing them without personal identifiers. Students generate their own code consisting of letters and numbers according to a predefined system, which they provide at each assessment.

All students are assessed at four measurement points. The intervention is delivered in four modules, each comprising a double class period (90 minutes), which are conducted in consecutive weeks. Assessments at the four measurement points (baseline, 1 month, 4 months, and 12 months) take place during one regular class period each (45 minutes per assessment, for a total of four 45-minute sessions).

To increase students' motivation to complete the questionnaires accurately, a prize draw [one iPad] is conducted among all participants. Written parental consent is obtained for participation in the draw. If a student has completed the questionnaires properly at a given assessment point, one entry bearing the corresponding pseudonym is created. Each student can therefore participate in the draw

with up to four entries. The codes of the winners are forwarded to the person who manages the key table. This person contacts the responsible teacher after the draw to inform the student of the prize. The students then contact the study coordinators themselves to receive the prize.

3.2.2 Sample size

For the study, all classes that receive the preventive intervention within the framework of the already approved PROTECTdissemination project are included. The target group of the prevention program and, at the same time, the study population therefore comprises 1,800 adolescents (900 per group: PROTECTtraining and PROTECTinfo) from 90 school classes (two groups per class, with at least 10 students per group) across 15 regional centers in Baden-Württemberg (six classes per center).
With this sample size, the results of the previously completed randomized controlled original PROTECT study can be validated under real-world conditions across different contexts, as well as separately by usage intensity and application form (computer gaming, communication, information seeking, and streaming).
Based on experiences from our previous studies, we estimate a dropout rate of 30%. This results in an expected total follow-up sample of N = 1,200 students (approximately 600 cases per group). This sample size also allows for the conduct of differential analyses by application form—(1) gaming, (2) communication and social networking, and (3) surfing and streaming—using structural equation models, for which at least N = 200 participants per group are required. With this sample size, a robust data basis for the investigation of interaction effects is provided.

3.2.3 Randomization

The assignment of students within each class to the two conditions (PROTECTtraining vs. PROTECTinfo) is carried out at random. Within each class, allocation is determined using a lottery procedure in a 1:1 ratio, stratified by gender.

3.2.4 Inclusion and Exclusion Criteria

Based on the typical age of onset in adolescence and the cognitive prerequisites required to work through the modules, PROTECT is aimed at students in secondary education. All students aged 12 years and older who agree to participate and whose parents have provided consent are eligible for inclusion. Participants may withdraw their consent at any time, without providing reasons. In order to examine the effectiveness of the PROTECT prevention program under real-world conditions, no additional exclusion criteria are applied.

3.2.5 Measures and Control Variables

Data collection is carried out using self-report questionnaires completed by participants at a total of four measurement points (for an overview of the measures and corresponding assessment time points, see Table 1). In addition to the measures used in the original study (Lindenberg, Halasy, & Schoenmaekers, 2017), the assessment includes additional scales to address relevant ancillary research questions. Some questionnaires were replaced for economic reasons and to optimize the study design.

Disorder-specific symptoms of gaming disorder and internet addiction, comorbid symptoms of other mental disorders (in particular depression and anxiety disorders), as well as additional problem areas, emotion regulation competencies, and subjective quality of life are assessed (dependent variables). In addition, general sociodemographic data (gender, age), academic performance, and the students' expected highest school-leaving qualification are collected.

Problematic gaming and internet use are assessed using the short version of the Compulsive Internet Use Scale (Short CIUS; Bischof, G., Bischof, A., Besser, B., & Rumpf, H. J., 2016) and the Computer Gaming Addiction Scale (CSAS; Rehbein, Baier et al., 2015). The Gaming Attitude Test (GAT; German: attitude scale toward computer gaming) is a questionnaire developed by our research group to assess attitudes toward computer gaming. A total score as well as two subscales—"preoccupation" (German: cognitive preoccupation; 8 items) and "beliefs" (German: beliefs; 7 items)—are calculated. In a pilot study (N = 200), reliability of the total scale ranged from .66 to .74, reliability of the "preoccupation" subscale from .76 to .84, and reliability of the "beliefs" subscale from .86 to .93. The two subscales are negatively correlated (−.27**).

The occurrence of Internet Gaming Disorder during the past 12 months is assessed using the Internet Gaming Disorder Scale (IGDS; Lemmens, Valkenburg, & Gentile, 2015). Problematic use of social media during the past 12 months is assessed using the Social Media Disorder Scale (SMDS; van den Eijnden, Lemmens, & Valkenburg, 2016). Problematic alcohol use during the past 12 months is assessed using the Alcohol Use Disorders Identification Test (AUDIT; Babor, Higgins-Biddle, Saunders, & Monteiro, 2001). Problematic cannabis use during the past 12 months is assessed using the Cannabis Abuse Screening Test (CAST; Legleye, Karila, Beck, & Reynaud, 2007). Problematic gambling behavior is operationalized using the Lie/Bet questionnaire (Johnson et al., 1997).

Personality dimensions are assessed using the short version of the Big Five Inventory for Children and Adolescents (BFI-K KJ; Kupper, Krampen, Rammstedt, & Rohrmann, 2019). Emotional problems, behavioral problems, attention problems, peer relationship problems, and prosocial behavior are assessed using the Strengths and Difficulties Questionnaire (SDQ; Goodman, Meltzer, & Bailey, 2003). Emotion regulation difficulties are assessed using the Difficulties in Emotion Regulation Scale – Short Form (DERS-SF; Kaufman et al., 2016). The level of social anxiety is assessed using the German version of the Social Interaction Anxiety Scale (SIAS; Stangier, Heidenreich, Beradi, Golbs, & Hoyer, 1999). Depressive symptoms are assessed using the Depression Screener for Teenagers (DesTeen; Pietsch et al., 2011). Performance anxiety is operationalized using the seventh scale of the Phobia Questionnaire for Children and Adolescents (PHOKI; Döpfner, Schnabel, Goletz, & Ollendick, 2006). The tendency to procrastinate is assessed

using the Procrastination Questionnaire for Students (PFS-4; Glöckner-Rist, Engberding, Höcker, & Rist, 2009).

The KIDSCREEN-27 (health-related quality of life questionnaire for children and adolescents; KIDSCREEN Group Europe, 2006) is used to assess various aspects of health-related quality of life in children and adolescents aged 8 to 18 years. The WHO-5 Well-Being Index (1998 version; Topp, Østergaard, Søndergaard, & Bech, 2015) is used to assess well-being in children and adolescents using a brief screening questionnaire.

Participants in both groups as well as the multipliers are additionally asked once to evaluate the modules.

Table 1: *Measures and Assessment Time Points.*

Measures	Pre-intervention	Post-intervention	4-Month-Follow-Up	12-Month-Follow-Up
Sociodemographics	x	-	-	-
Academic performance	x	-	-	-
Short CIUS	x	x	x	x
CSAS	x	x	x	x
GAT	x	x	x	x
IGDS	x	x	x	x
SMDS	x	x	x	x
AUDIT	x	-	-	x
CAST	x	-	-	x
Lie/bet Questionnaire	x	-	-	x
BFI-K KJ	x	-	-	-
SDQ	x	x	x	x
DERS-SF	x	x	x	x
SIAS	x	x	x	x
DesTeen	x	x	x	x
PHOKI Performance Anxiety	x	x	x	x
PFS-4	x	x	x	x
KIDSSCREEN-27	x	x	x	x
WHO-5	x	x	x	x
Student Evaluation	-	x	-	-
Multiplier Evaluation	-	x	-	-

3.2.6 Study design

he PROTECTconfirm study is planned as a two-arm, multicenter, prospective longitudinal study designed to examine the preventive effectiveness of two variants of the PROTECT prevention program (PROTECTtraining and PROTECTinfo), which differ in their intensity. This approach is intended to account for the heterogeneity of the target population.

All study participants are assessed at four measurement points (see Section 3.2.5 for the measures): at project start, after completion of the intervention, after 4 months, and after 12 months. Allocation of adolescents to the groups (independent variable) is carried out by randomization using a lottery procedure conducted by the multipliers. Figure 1 schematically illustrates the assessment time points.

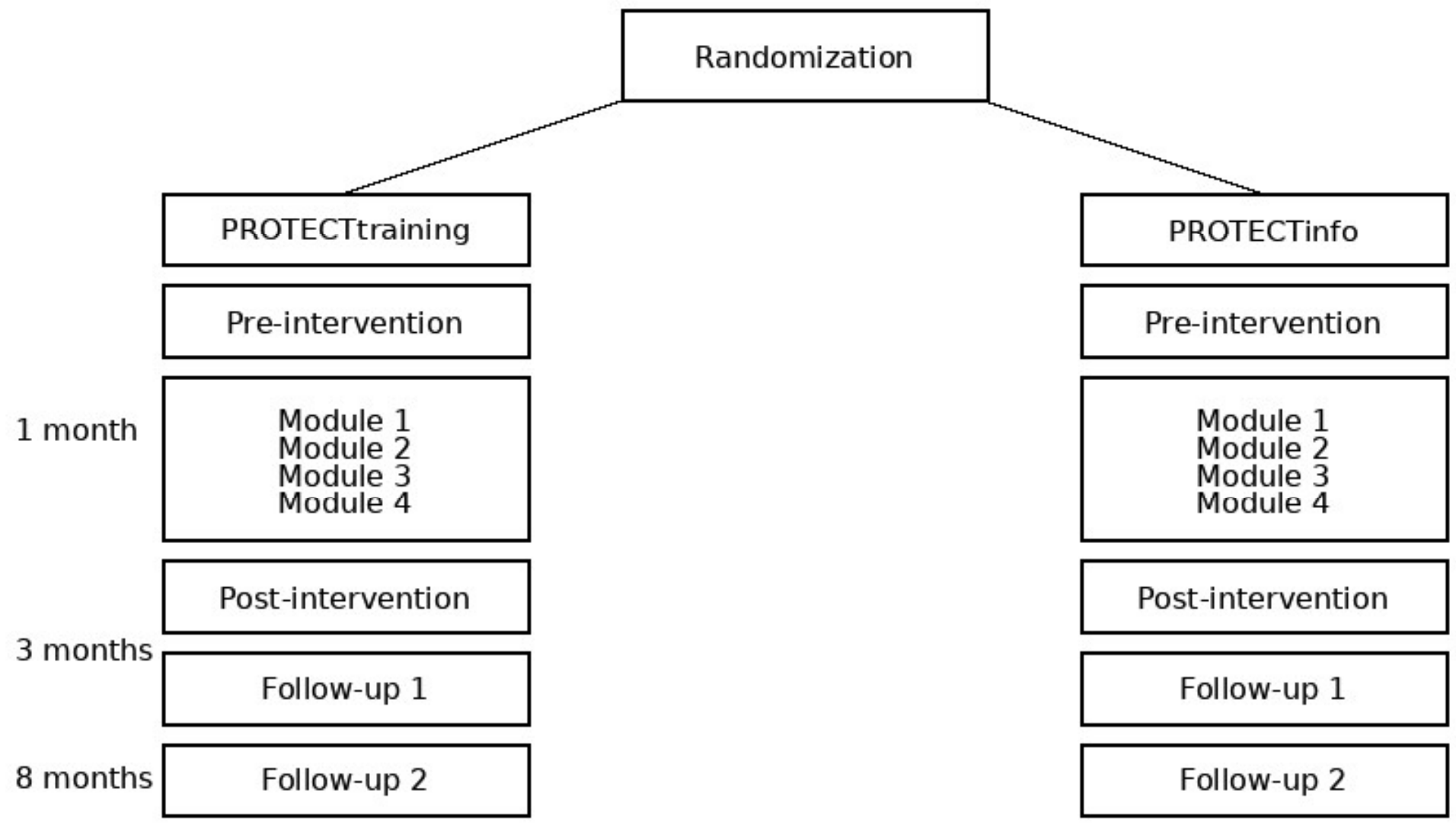

Figure 1. Schematic illustration of the study design and assessment schedule.

3.2.7 Statistical Analyses / Data Analysis

nalyses of changes in symptoms over time are conducted using hierarchical linear modeling. Analyses of interaction effects, mediators, and moderators are carried out using structural equation models. Mediator and moderator analyses are used to examine interaction effects and mechanisms of action of the preventive intervention, as well as differential effectiveness under varying contextual conditions (regional differences, differences by school type, and sociodemographic differences).

In addition, health economic analyses (cost-utility analyses, CUA) are performed. For this purpose, participants' subjectively assessed quality of life is used to determine quality-adjusted life years (QALYs). QALYs represent a key index in health economics for conducting cost-utility analyses (CUA).

3.2.8 Work Plan and Timeline With Milestones

The study spans a period of three years and follows the timeline outlined below.

Month	Main Activity	Milestone
**1 – 4**	Preparation phase	M 4: All approvals obtained, including positive ethics approval
**1 – 13**	Training of key personnel	M 13: Training completed
**1 – 18**	Recruitment phase of schools/participants	M 12: 50% 50% recruited<br>M 18: Recruitment completed
**5 – 19**	Intervention	M 19: Interventions completed
**8 – 31**	Follow-Up Assessment	M 31: Follow-Up Assessments completed abgeschlossen
**33 – 36**	Data analysis and publication	M 36: Manuscript submitted

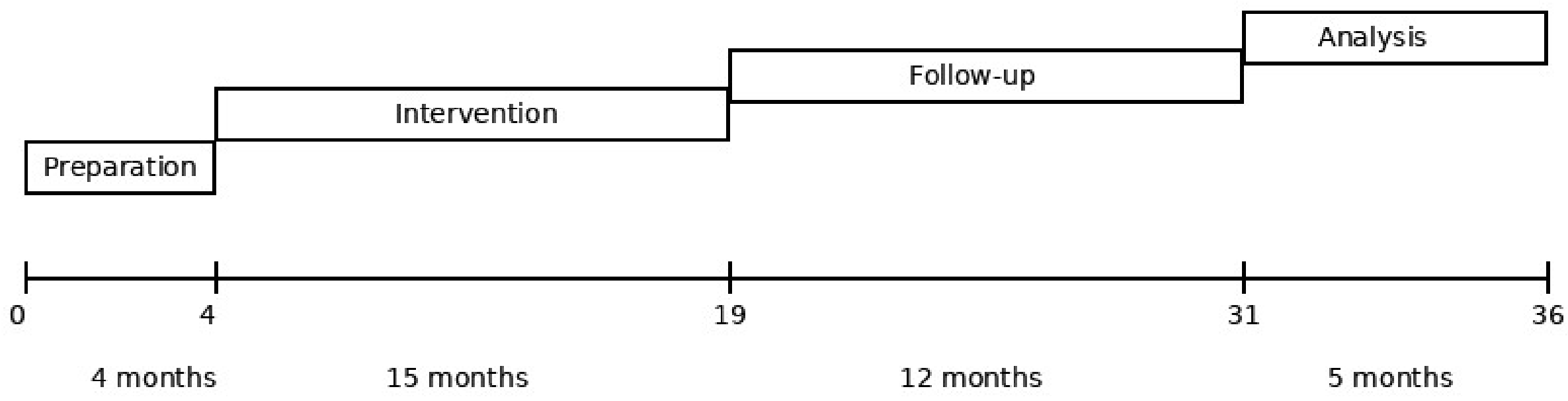

Figure 2. Schematic illustration of the assessment schedule.

3.3 Studies Involving Human Participants

3.3.1 Safety Measures for Study Participants

- Both PROTECT interventions (PROTECTtraining and PROTECTinfo) are delivered by appropriately trained personnel to ensure the quality of the preventive intervention.
- As the project is conducted in cooperation with the Ministry of Social Affairs and Integration of Baden-Württemberg and coordination is also carried out via the addiction commissioners

of the regional centers, timely referral of at-risk students to routine care can be ensured by these bodies if necessary.

- Participating schools, parents, and students receive an information letter providing details about the project, its procedures, and contact options for the study coordinators.
- During each data collection and intervention session, trained professionals (addiction specialists, psychologists) are present on site, who are also trained to manage crisis situations.

3.3.2 Criteria for Study Discontinuation

No risks associated with participation in the preventive brief intervention are identifiable. The study will be discontinued if it becomes apparent that participation in the prevention program results in significant disadvantages for participants in either group.

3.3.3 Ethical and Legal Considerations

The study is conducted in accordance with the current version of the Declaration of Helsinki.

Participation in the study is voluntary and takes place only with the consent of the teaching staff (following integration of the prevention program into the curricular prevention plan), as well as with the consent of parents and students. Consent may be withdrawn at any time, without providing reasons and without any disadvantages for further care.

Participants and parents are informed in writing prior to study initiation about the nature and scope of the planned study, in particular about potential benefits for health and any possible risks. Consent is documented by parents' signatures on the informed consent form. Students who are of legal age sign the consent form themselves. Upon withdrawal from the study, already collected data may be destroyed upon request.

Pseudonymization of data use is guaranteed at all times. The study protocol is submitted to the ethics committee for review prior to study initiation. No participants are enrolled before written approval from the ethics committee has been obtained. The names of participants and all other confidential information are subject to medical confidentiality and the provisions of the German Federal Data Protection Act (Bundesdatenschutzgesetz, BDSG). Participant data are disclosed, if at all, only in anonymized form. Third parties are not granted access to original records.

3.4 Data Protection

Prior to the start of data collection, approval for the present study protocol is obtained from the ethics committee of the Institute of Psychology of Goethe University Frankfurt am Main. The ethical and professional regulations of the German Psychological Society (DGP) and the German Association of Psychologists (BDP) are observed, as well as the German Federal Data Protection Act (BDSG) or the respective state data protection laws and the EU GDPR. All of this applies in particular to the provision of information to study participants and their voluntary participation, compliance with

confidentiality obligations, and compliance with data protection with regard to data collection, processing, and use.

The determination of the amount of data to be processed was carried out according to the principle of data minimization. The data to be processed are personal data that are available in pseudonymized form. The data are collected by M.Sc. Psych. Sarah Hofmann, M.Sc. Psych. Sonja Kewitz, and Dipl.-Psych. Katharina Leo and processed as follows:

The name of the student as well as their date of birth, school, and class are collected on the parents' consent form. With the help of these data, a pseudonym is generated. The consent forms containing the personal data are securely stored at a central location at the Institute of Psychology, Department of Child and Adolescent Psychotherapy, in lockable cabinets that are accessible exclusively to the study coordinators. Spatially separate from the consent forms, the completed pseudonymized questionnaires are stored in a lockable room in the Department of Child and Adolescent Psychotherapy. An electronic list is created that records the pseudonym and name (unblinding list). This list is stored in encrypted form locally on the computers of the study coordinators. Only the study coordinators have knowledge of the encryption procedure. Entry of the pseudonymized data into the protected database network of the Department of Child and Adolescent Psychotherapy is carried out by the study coordinators and, if applicable, by (student) research assistants. The collection and storage of the data in pseudonymized form is carried out in order to be able to identify and delete individual data in the event of withdrawal from study participation, as well as to be able to assign datasets from four measurement time points to one another. The duration of data storage is ten years. After expiry of this period, all physical data are properly destroyed by a certified specialist company. The unblinding list is deleted after publication of the data.

If, due to the COVID-19 pandemic or due to student absences, online surveys are used, exclusively pseudonymized data are collected using the Unipark program. The corresponding link for data collection is distributed to students via teachers.

The results of the study are expected to be published in psychological scientific journals without any possibility of reference to personal identifying information.

References

American Psychiatric Association (2013). *Diagnostic and Statistical Manual of Mental Disorders, fifth edition* (5th ed.). Washington, D.C.: American Psychiatric Association.

Anderson, E. L., Steen, E., & Stavropoulos, V. (2017). Internet use and problematic internet use: a systematic review of longitudinal research trends in adolescence and emergent adulthood. *International Journal of Adolescence and Youth*, *22*(4), 430–454. https://doi.org/10.1080/02673843.2016.1227716

Babor, T.F., Higgins-Biddle, J.C., Saunders, J.B. and Monteiro, M.G. (2001). The Alcohol Use Disorders Identification Test (AUDIT) manual: Guidelines for use in primary care. 2nd Edition, Department of Mental Health and Substance Dependence. (WHO/MSD/MSB/01.6a, 4-32.). Retrieved from https://apps.who.int/iris/handle/10665/67205

Bischof, G., Bischof, A., Besser, B. & Rumpf, H. J. (2016). *Problematische und pathologische Internetnutzung: Entwicklung eines Kurzscreenings (PIEK). Abschlussbericht an das Bundesministerium für Gesundheit, Lübeck*. Universität zu Lübeck, Klinik für Psychiatrie und Psychotherapie.

Carli, V., Durkee, T., Wasserman, D., Hadlaczky, G., Despalins, R., Kramarz, E., . . . Kaess, M. [M.] (2013). The association between pathological internet use and comorbid psychopathology: A systematic review. *Psychopathology*, *46*(1), 1–13. https://doi.org/10.1159/000337971

Döpfner, M., Schnabel, M., Goletz, H., & Ollendick, T. (2006). *Phobiefragebogen für Kinder und Jugendliche: PHOKI*. Göttingen: Hogrefe.

Glöckner-Rist, A., Engberding, M., Höcker, A., & Rist, F. (2009). *Prokrastinationsfragebogen für Studierende (PFS).* https://doi.org/10.6102/zis140

Goodman, R., Meltzer, H., & Bailey, V. (2003). The Strengths and Difficulties Questionnaire: a pilot study on the validity of the self-report version. *International Review of Psychiatry (Abingdon, England)*, *15*(2), 173–177. https://doi.org/10.1080/0954026021000046137

Gürtler, D., Rumpf, H.-J., Bischof, A., Kastirke, N., Meerkerk, G.-J., John, U., & Meyer, C. (2015). Psychometrische Eigenschaften und Normierung der deutschen Version der Compulsive Internet Use Scale (CIUS). *Diagnostica*, *61*(4), 210–221. https://doi.org/10.1026/0012-1924/a000127

Ho, R. C., Zhang, M. W. B., Tsang, T. Y., Toh, A. H., Pan, F., Lu, Y., . . . Mak, K.-K. (2014). The association between internet addiction and psychiatric co-morbidity: A meta-analysis. *BMC Psychiatry*, *14*, 183. https://doi.org/10.1186/1471-244X-14-183

Johnson, E. E., Hamer, R., Nora, R. M., Tan, B., Eisenstein, N., & Engelhart, C. (1997). The Lie/Bet Questionnaire for screening pathological gamblers. *Psychological Reports*, *80*(1), 83–88. https://doi.org/10.2466/pr0.1997.80.1.83

Kaufman, E. A., Xia, M., Fosco, G., Yaptangco, M., Skidmore, C. R., & Crowell, S. E. (2016). The Difficulties in Emotion Regulation Scale Short Form (DERS-SF): Validation and Replication in Adolescent and Adult Samples. *Journal of Psychopathology and Behavioral Assessment*, *38*(3), 443–455. https://doi.org/10.1007/s10862-015-9529-3

KIDSCREEN Group Europe (2006). *The KIDSCREEN questionnaires: Quality of life questionnaires for children and ado-lescents handbook.* Lengerich.

King, D. L., Delfabbro, P. H., Doh, Y. Y., Wu, A. M. S., Kuss, D. J., Pallesen, S., . . . Sakuma, H. (2018). Policy and prevention approaches for disordered and hazardous gaming and internet use: An International perspective. *Prevention Science : The Official Journal of the Society for Prevention Research*, *19*(2), 233–249. https://doi.org/10.1007/s11121-017-0813-1

Ko, C. H., Yen, J. Y., Yen, C. F., Chen, C. S., & Chen, C. C. (2012). The association between Internet addiction and psychiatric disorder: A review of the literature. *European Psychiatry : The Journal of the Association of European Psychiatrists*, *27*(1), 1–8. https://doi.org/10.1016/j.eurpsy.2010.04.011

Kupper, K., Krampen, D., Rammstedt, B., & Rohrmann, S. (2019). Kurzversion des Big Five Inventory für Kinder und Jugendliche (BFI-K KJ). *Diagnostica*, *65*(2), 86–96. https://doi.org/10.1026/0012-1924/a000216

Legleye, S., Karila, L., Beck, F., & Reynaud, M. (2007). Validation of the CAST, a general population Cannabis Abuse Screening Test. *Journal of Substance Use*, *12*(4), 233–242. https://doi.org/10.1080/14659890701476532

Lemmens, J. S., Valkenburg, P. M., & Gentile, D. A. (2015). The Internet Gaming Disorder Scale. *Psychological Assessment*, *27*(2), 567–582. https://doi.org/10.1037/pas0000062

Lindenberg, K., & Forschungsgruppe Computerspiel- und Internetabhängigkeit (2018). *PROTECT: Ein Gruppentraining bei hohem Internetkonsum*. Heidelberg: Hochschulinterner Druck.

Lindenberg, K., Halasy, K., & Schoenmaekers, S. (2017). A randomized efficacy trial of a cognitive-behavioral group intervention to prevent Internet Use Disorder onset in adolescents: The PROTECT study protocol. *Contemporary Clinical Trials Communications*, *6*, 64–71. https://doi.org/10.1016/j.conctc.2017.02.011

Lindenberg, K., Halasy, K., Szász-Janocha, C., & Wartberg, L. (2018). A phenotype classification of Internet Use Disorder in a large-scale high-school study. *International Journal of Environmental Research and Public Health*, *15*(4). https://doi.org/10.3390/ijerph15040733

Lindenberg, K., Szász-Janocha, C., Schoenmaekers, S., Wehrmann, U., & Vonderlin, E. (2017). An analysis of integrated health care for Internet Use Disorders in adolescents and adults. *Journal of Behavioral Addictions*, *6*(4), 579–592. https://doi.org/10.1556/2006.6.2017.065

Petersen, K.-U., & Thomasius, R. (2010). Beratungs- und Behandlungsangebote zum pathologischen Internetgebrauch in Deutschland: Endbericht an das Bundesministerium für Gesundheit (BMG). Retrieved from https://www.bundesgesundheitsministerium.de/fileadmin/Dateien/5_Publikationen/Drogen_und_Sucht/Berichte/Studie_Beratungsangebote_pathologischer_Internetgebrauch.pdf

Pietsch, K., Allgaier, A.-K., Frühe, B., Rohde, S., Hosie, S., Heinrich, M., & Schulte-Körne, G. (2011). Screening for depression in adolescent paediatric patients: Validity of the new Depression Screener for Teenagers (DesTeen). *Journal of Affective Disorders*, *133*(1-2), 69–75. https://doi.org/10.1016/j.jad.2011.03.026

Rehbein, F., Baier, D., Kleinmann, M., & Mößle, T. [T.] (2015). *CSAS: Computerspielabhängigkeitsskala: Ein Verfahren zur Erfassung der Internet Gaming Disorder nach DSM-5*. Göttingen: Hogrefe.

Rehbein, F., Kliem, S., Baier, D. [Dirk], Mößle, T. [Thomas], & Petry, N. M. (2015). Prevalence of Internet gaming disorder in German adolescents: Diagnostic contribution of the nine DSM-5 criteria in a state-wide representative sample. *Addiction (Abingdon, England)*, *110*(5), 842–851. https://doi.org/10.1111/add.12849

Stangier, U., Heidenreich, T., Beradi, A., Golbs, U., & Hoyer, J. (1999). Die Erfassung sozialer Phobie durch Social Interaction Anxiety Scale (SIAS) und die Social Phobia Scale (SPS). *Zeitschrift Für Klinische Psychologie*, *28*(1), 28–36. https://doi.org/10.1026//0084-5345.28.1.28

Statistisches Bundesamt (2017). Gesundheit: Krankheitskosten: 2015. Retrieved from https://www.destatis.de/DE/Publikationen/Thematisch/Gesundheit/Krankheitskosten/KrankheitskostenJahr2120721159004.pdf?__blob=publicationFile

Strittmatter, E., Kaess, M. [Michael], Parzer, P., Fischer, G., Carli, V., Hoven, C. W. [Christina W.], . . . Wasserman, D. [Danuta] (2015). Pathological Internet use among adolescents: Comparing gamers and non-gamers. *Psychiatry Research*, *228*(1), 128–135. https://doi.org/10.1016/j.psychres.2015.04.029

Throuvala, M. A., Griffiths, M. D., Rennoldson, M., & Kuss, D. J. (2019). School-based prevention for adolescent internet addiction: Prevention is the key. A systematic literature review. *Current Neuropharmacology*, *17*(6), 507–525. https://doi.org/10.2174/1570159X16666180813153806

Topp, C. W., Østergaard, S. D., Søndergaard, S., & Bech, P. (2015). The WHO-5 Well-Being Index: A systematic review of the literature. *Psychotherapy and Psychosomatics*, *84*(3), 167–176. https://doi.org/10.1159/000376585

Van den Eijnden, R. J.J.M., Lemmens, J. S., & Valkenburg, P. M. (2016). The Social Media Disorder Scale. *Computers in Human Behavior*, *61*, 478–487. https://doi.org/10.1016/j.chb.2016.03.038

Vondráčková, P., & Gabrhelík, R. (2016). Prevention of Internet addiction: A systematic review. *Journal of Behavioral Addictions*, *5*(4), 568–579. https://doi.org/10.1556/2006.5.2016.085

Wartberg, L., Kriston, L., Kammerl, R., Petersen, K.-U., & Thomasius, R. [Rainer] (2015). Prevalence of pathological internet use in a representative German sample of adolescents: Results of a latent profile analysis. *Psychopathology*, *48*(1), 25–30. https://doi.org/10.1159/000365095

Wartberg, L., Kriston, L., & Thomasius, R. [Rainer] (2017). The prevalence and psychosocial correlates of Internet Gaming Disorder. *Deutsches Arzteblatt International*, *114*(25), 419–424. https://doi.org/10.3238/arztebl.2017.0419

Wartberg, L., Kriston, L., Zieglmeier, M., Lincoln, T., & Kammerl, R. (2018). A longitudinal study on psychosocial causes and consequences of Internet gaming disorder in adolescence. *Psychological Medicine*, *49*(2), 287–294. https://doi.org/10.1017/S003329171800082X

Wölfling, K., Jo, C., Bengesser, I., Beutel, M. E., & Müller, K. W. (2013). *Computerspiel- und Internetsucht: Ein kognitiv-behaviorales Behandlungsmanual. Störungsspezifische Psychotherapie*. Stuttgart: Kohlhammer.

World Health Organization (2018). ICD-11: International Classification of Diseases 11th Revision. Retrieved from https://icd.who.int/dev11/l-m/en
