# Supplementary material for: Cognitive Behavioral Therapy vs Media Literacy for Prevention of Gaming Disorder and Unspecified Internet Use Disorder: A Randomized Clinical Trial
Source: JAMA Netw Open. 2026 Sep 4;9(9):e2631942. doi: 10.1001/jamanetworkopen.2026.31942 (PMC13545672; doi:10.1001/jamanetworkopen.2026.31942)
Supplement: Supplementary file 2 [file jamanetwopen-e2631942-s002.pdf]

Supplementary Online Content

Lindenberg K, Kewitz S, Neumann I, et al. Cognitive behavioral therapy vs media literacy for prevention of gaming disorder and unspecified internet use disorder: a randomized clinical trial. *JAMA Netw Open.* 2026;9(9):e2631942.
doi:10.1001/jamanetworkopen.2026.31942

eMethods.

eTable 1. Measures and Assessment Time Points

eTable 2. Completer Analysis. Estimated Marginal Means, Observed *SD*s, and Effect Sizes of the Primary Outcome

eTable 3. Per-Protocol Analysis. Estimated Marginal Means, Observed *SD*s, and Effect Sizes of the Primary Outcome

eTable 4. Secondary Outcomes. Estimated Marginal Means, Observed *SD*s, and Effect Sizes of Secondary Outcomes After Multiple Imputation (Intention-to-Treat Analyses)

eTable 5. Responder Analysis. Frequencies of Reliable Improvement From Pretest to 12-Months Follow-Up on Primary (Observed Data)

eTable 6. Negative Effects. Frequencies of Reliable Deterioration From Pretest to 12-Months Follow-Up on Primary Outcome (Observed Data)

eTable 7. Adherence. Descriptive Statistics of Adherence Ratings

eReferences.

This supplementary material has been provided by the authors to give readers additional information about their work.

© 2026 Lindenberg K et al. *JAMA Network Open.*

eMethods.

This supplemental section provides additional information on trial registration, ethics, design and setting, eligibility criteria, interventions, outcome measures, randomization and allocation concealment, blinding, procedure, data protection, protocol changes, sample size estimation, and statistical analysis.

Study Protocol, Trial Registration, Ethics and Approval by the Ministry of Education

All hypotheses were preregistered in the study protocol submitted on July 30, 2020, prior to the beginning of enrollment. The study was registered in the German Clinical Trials Register (DRKS00033989) on April 3, 2024, prior to completion of data collection.
The trial was conducted in accordance with the Declaration of Helsinki and applicable data protection laws (EU General Data Protection Regulation and Federal Data Protection Act). The study was approved by the Ethics Committee of the Department of Psychology and Sports Sciences at Goethe University Frankfurt (approval No. 2020-48) and authorized by the Ministry of Education, Youth and Sports of Baden-Württemberg (AZ 31-6499.20/1334/1) prior to data collection.

Participants

Participants were students aged 11–18 years recruited from 92 classes across 44 secondary schools in the federal state of Baden-Württemberg, Germany. Schools were selected to ensure representation across school types (academic, intermediate, and lower-track secondary schools) as well as urban and rural regions. Recruitment took place from September 2020 to July 2023, with follow-up assessments conducted through July 2024. Participating schools agreed to integrate the interventions into their regular curriculum.
Of the 92 classes comprising 2,198 students in which PROTECTtraining and PROTECTinfo were delivered in 184 half-class groups, 1,792 students from 90 classes participated in the study (81.6% participation rate). Two classes received the interventions but did not participate in the study because teachers declined study participation for organizational reasons. Among the 90 classes with study participants, class sizes ranged from 12 to 32 students, resulting in intervention group sizes of 6–16 students per half class. Because participation in the study required written parental consent and student assent, the number of study participants per half-class intervention group ranged from 2 to 16.

Allocation Concealment and Blinding

Within each of the 90 participating classes, students were individually randomized after written informed consent had been obtained, using a 1:1 allocation to PROTECTtraining or PROTECTinfo. Randomization was conducted by study team members responsible for live supervision, who had no personal contact with students and no influence on the prespecified, class-stratified alternating allocation sequence (0–1–0–1 or 1–0–1–0); the starting condition was determined at random. Teachers collecting consent forms and facilitators delivering the interventions were unaware of the starting condition. Allocation was implemented by supervisory study staff, who documented the sequence either on the class list (if available in advance) or on the questionnaire stack in sequential order, which was then distributed according to the seating arrangement. Personnel involved in enrollment and assignment could not influence the allocation sequence.

Because of the nature of the interventions, participants, facilitators, supervisors, and data analysts were aware of group assignment; however, participants and facilitators were not informed that the media-literacy condition functioned as the control group or of the study's superiority hypothesis.

Interventions

Both interventions were manualized and comprised four weekly 90-minute group sessions delivered to half-class-size groups during regular school hours. The PROTECTtraining manual is published as book [1], the PROTECTinfo manual can be received upon request to protect@apw-heidelberg.de.

PROTECTtraining. PROTECTtraining is a cognitive-behavioral intervention targeting maladaptive cognitions, dysfunctional coping, emotion dysregulation, and impulse-control deficits.

© 2026 Lindenberg K et al. *JAMA Network Open.*

Core components include (1) psychoeducation on addictive mechanisms and reward processes in digital media use, (2) identification and cognitive restructuring of dysfunctional thoughts, (3) behavioral activation and problem-solving techniques to promote healthy alternative behaviors, and (4) emotion regulation and coping skills training. Sessions combine case examples, brief theoretical input, interactive exercises, role-plays, and homework assignments. The manual is available at Lindenberg et al., 2020[1].

PROTECTinfo. PROTECTinfo is a media-literacy intervention matched in duration, group format, and delivery mode. It focuses on (1) mobile phone use, apps, and privacy, (2) communication in physical vs virtual environments, (3) risks and benefits of video gaming, and (4) cyberbullying and human rights. Unlike PROTECTtraining, PROTECTinfo does not include cognitive-behavioral or emotion-regulation components beyond psychoeducation. The manual is available at doi:10.23668/PSYCHARCHIVES.22351.

Training of local professionals

Facilitator training was funded by the Ministry of Social Affairs, Health and Integration of Baden-Württemberg, which announced participation in the training program to prevention professionals across the entire federal state. This statewide recruitment strategy aimed to reach professionals working in both urban and rural regions and collaborating with different school types (academic-track, intermediate-track, and lower-track secondary schools), thereby supporting equitable access to school-based preventive services. The funding enabled the establishment of a structured dissemination framework within the region.

Within this framework, a non-profit organization (Academy for Prevention Science, www.apw-heidelberg.de) was founded to coordinate training and implementation. A total of 111 local professionals (including teachers, school psychologists, social workers, youth counselors, and addiction counselors) were trained as certified PROTECT facilitators across multiple regional centers in Baden-Württemberg. Training was delivered in nine standardized four-day workshops, comprising two days of PROTECTtraining and two days of PROTECTinfo. Workshops included theoretical instruction, demonstrations, and structured role-plays.

Following completion of the workshops, facilitators conducted their first intervention groups under live supervision by expert psychologists, allowing for supervised practice and feedback on protocol adherence. Ongoing live supervision by certified experts was provided throughout the study to ensure intervention fidelity and implementation quality.

Procedure

To evaluate intervention effectiveness under real-world conditions (i.e., delivery by trained local professionals), additional funding was obtained from the Federal Ministry of Education and Research, enabling implementation of the PROTECTconfirm study. Following completion of their theoretical training, facilitators recruited participating schools (n = 44) and classes (n = 92). Recruitment procedures included the distribution of standardized information materials to school principals, parents, and students. The target population comprised students from grade 6 onwards. Implementation took place under routine school conditions. Participating schools integrated PROTECTtraining and PROTECTinfo into the regular school curriculum. All students in participating classes attended one of the two intervention formats, which were delivered in small groups corresponding to half-class size. While intervention groups always reflected half-class sizes (maximum class size in Germany: 32 students; maximum intervention group size: 16 students), participation in the study, defined as participation in the four waves of data collection, was voluntary and required written informed consent from parents as well as student assent. Consequently, the number of students providing study data per intervention group ranged from 0 (in 2 classes) to 16. Assessments were conducted during regular school hours. Data were collected using standardized paper-and-pencil questionnaires or online surveys administered via Unipark.

Anonymization. Data were collected and stored in pseudonymized form, with personally identifying information kept separately in locked files accessible only to authorized staff. Participants could withdraw from the study at any time without disadvantage, and their data would be deleted upon request. The name of the student as well as their date of birth, school, and class were collected on the parents' consent form. With the help of these data, a pseudonym was generated. The consent forms

© 2026 Lindenberg K et al. *JAMA Network Open.*

containing the personal data were securely stored at a central location at the Institute of Psychology, in lockable cabinets that are accessible exclusively to the study coordinators. Spatially separate from the consent forms, the completed pseudonymized questionnaires were stored in a lockable room. An electronic list was created that recorded the pseudonym and name (unblinding list). This list was stored in encrypted form locally on the computers of the study coordinators. Only the study coordinators had knowledge of the encryption procedure. Entry of the pseudonymized data into the protected database network of the Department of Child and Adolescent Psychotherapy is carried out by the study team. The collection and storage of the data in pseudonymized form was carried out in order to be able to identify and delete individual data in the event of withdrawal from study participation, as well as to be able to assign datasets from four measurement time points to one another.

Changes to Trial Protocol. The interventions were originally intended to be delivered on site in half-class–sized groups. During COVID-19–related school closures, training sessions were delivered during home schooling in live, video-based group formats replicating in-person procedures. This applied to 4 out of 92 groups of PROTECTinfo and 4 out of 92 groups of PROTECTtraining. Following approval of an amendment by the ethics committee, a measure of problematic pornography use and a measure of problematic shopping behavior were added to all T4 assessments, as prevalence data for these behaviors in minors are currently lacking and these domains appeared to be of high scientific relevance given the growing body of evidence in adult populations.

Measures and Outcomes

Sociodemographic and internet usage measures

Age, gender, academic level and academic performance in German and Mathematics were assessed at baseline. Average daily time spent online on gaming and time spent online on social media were assessed and computed as follows: (5*average time on weekdays + 2*average time on weekends)/7. Reported time spent exceeding 12 hours per day was truncated at 12 hours.

Primary Outcome

Symptom severity of Internet Gaming and unspecified Internet Use Disorders. The primary outcome was symptom severity of Internet Gaming and unspecified Internet Use Disorders, assessed with a modified Video Game Dependency Scale [2,3], as in our previous study [4]. In its original version, this 18-item questionnaire measures 9 criteria of Internet Gaming Disorder according to DSM-5 frameworks (preoccupation, withdrawal, tolerance, loss of control, loss of interests, continued use despite problems, deception, escape, and jeopardizing significant relationships or opportunities), with each criterion assessed by two items. The scale therefore provides both a continuous measure of symptom severity and a criterion-based scoring approach aligned with the DSM-5 framework. Identical to the preceding PROTECT trial, we modified the instrument with permission by the publisher to capture both gaming-related and other forms of Internet Use Disorder (i.e., social media, streaming, online shopping or online pornography), following the approach of Lindenberg et al. (2022). We adapted the CSAS items to cover both Internet Gaming or Unspecified Internet Use Disorder in a common score (e.g., item 1: "Even when I am not gaming/online, I think about online gaming/the Internet" for preoccupation) with permission by the publisher. Items are rated on a 4-point Likert scale (0 = strongly disagree, 1 = somewhat disagree, 2 = somewhat agree, 3 = strongly agree), yielding a total score range from 0 to 54, with higher scores indicating greater symptom severity. The modified scale demonstrated excellent internal consistency (Cronbach $\alpha > .90$) in the preceding PROTECT trial. The primary analysis metric was change in total score from baseline to 12-month follow-up.

The modified Video Game Dependency Scale was additionally used to classify students at high-risk to be included in the indicated prevention subsample. Therefore, we used the scale's categorical scoring algorithm, which identifies fulfillment of individual DSM-5 criteria for Internet Gaming Disorder and unspecified Internet Use Disorders. A criterion is met, if at least one of the items has been rated with 3 (strongly agree). The instrument is therefore more conservative than questionnaires that assess each DSM-5 criterion with only a single item and dichotomous item response format. The inclusion criterion for the high-risk subsample in our study was the presence of two or more DSM-5

© 2026 Lindenberg K et al. *JAMA Network Open.*

criteria at baseline. This cut-off is proposed by the authors of the CSAS to define a group at-risk of developing a disorder [3].

Secondary Outcomes

General Internet Use Disorders. Short Compulsive Internet Use Scale (CIUS). The Compulsive Internet Use Scale [5] was used in its 5-item short version [6]. The S-CIUS consists of 5 items rated on a 5-point Likert scale from 0 (never) to 4 (very often), yielding a sum score range of 0–20, with higher scores indicating more severe compulsive internet use.

Problematic Gaming. Problematic Gaming was assessed by three measures capturing different concepts and aspects. The DSM-5-based Internet Gaming Disorder Scale [7] is a 9-item self-report instrument assessing DSM-5 criteria for Internet Gaming Disorder. Items are answered dichotomously (0 = no, 1 = yes), yielding a sum score range of 0–9, with higher scores indicating greater symptom severity. In accordance with DSM-5 diagnostic recommendations, endorsement of ≥5 criteria is typically used as a clinical cutoff indicating probable Internet Gaming Disorder. In the validation study, the scale demonstrated good internal consistency and strong criterion validity against clinical diagnosis. The ICD-11-based Gaming Disorder Test [8]. The Gaming Disorder Test is a 4-item self-report measure assessing Gaming Disorder according to the ICD-11 diagnostic framework. Items are rated on a 5-point Likert scale from 1 (never) to 5 (very often), resulting in a sum score range of 4–20, with higher scores indicating greater symptom severity. A commonly used clinical cutoff is endorsement of all four criteria (i.e., rating ≥4 on each item), corresponding to the ICD-11 diagnostic requirement that all core symptoms be present. In the validation study, the GDT demonstrated excellent internal consistency as well as strong construct and criterion validity across international samples. The Gaming Attitude Test GAT; [9] is a self-report measure designed to assess adolescents' cognitive and evaluative attitudes toward gaming. The likert-scaled items (response format: 1–5) result in two subscales (trivializing and preoccupation), with higher scores indicating more preoccupation with or trivializing attitudes toward gaming. Because the GAT is a construct measure of attitudes rather than a diagnostic screening tool, no clinical cutoff score has been established. In the validation study, the scale demonstrated good internal consistency (Cronbach's α values in the acceptable-to-good range across subscales) and satisfactory construct validity.

Problematic Social Media Use. Problematic social media use was assessed using the Social Media Disorder Scale [10], a 9-item self-report instrument based on DSM-5 criteria for behavioral addiction. Items are answered dichotomously (0 = no, 1 = yes), yielding a sum score range of 0–9, with higher scores indicating greater symptom severity. Endorsement of ≥5 criteria is typically used as a clinical cutoff indicating probable Social Media Disorder. In the validation study, the SMDS demonstrated good internal consistency as well as strong construct and criterion validity.

General psychopathology. General psychopathology was assessed using the self-report version of the Strengths and Difficulties Questionnaire [11]. The SDQ comprises 25 items rated on a 3-point scale (0 = not true, 1 = somewhat true, 2 = certainly true). It yields five subscales (Emotional Symptoms, Conduct Problems, Hyperactivity/Inattention, Peer Problems, Prosocial Behavior). The Total Difficulties Score is calculated by summing the first four problem subscales (excluding Prosocial Behavior), resulting in a score range of 0–40, with higher scores indicating greater psychopathology. Established normative cutoffs classify Total Difficulties scores as normal (0–15), borderline (16–19), and abnormal (20–40) in adolescent self-report samples. In validation studies, the self-report SDQ demonstrated acceptable internal consistency for the total difficulties score, with lower reliability for certain subscales, and good convergent validity with established clinical measures.

ADHD. ADHD symptoms were assessed using the Conners-3 ADHD Index [12], a brief screening scale derived from the Conners 3rd Edition rating system. The index consists of 10 items rated on a 4-point Likert scale (0 = not at all to 3 = fully agree), yielding a raw score range of 0–30, with higher scores indicating greater ADHD symptom severity. Validation studies of the Conners-3 ADHD Index report good internal consistency and strong discriminative validity for identifying ADHD cases.

Depression. Depressive symptoms were assessed using the Depression Screener for Teenagers [13], a brief self-report screening instrument for depressive symptoms in adolescents. The DesTeen consists of 10 items rated on a 3-point Likert scale (0–2), yielding a total score range of 0–20, with higher scores indicating greater depressive symptom severity. Validation analyses identified a cutoff score ≥12 as optimal for detecting clinically relevant depression, demonstrating good sensitivity and

© 2026 Lindenberg K et al. *JAMA Network Open.*

specificity in adolescent samples. In the validation study, the scale showed good internal consistency as well as strong convergent validity with established depression measures.

Social Anxiety. Social anxiety was assessed using the German version of the Social Interaction Anxiety Scale [14]. The SIAS comprises 20 items assessing anxiety in dyadic and group social interactions. Items are rated on a 5-point Likert scale (0–4), yielding a total score range of 0–80, with higher scores indicating greater social interaction anxiety. Validation studies suggest a clinical cutoff of ≥34 to indicate probable social anxiety disorder. In the original validation study, the SIAS demonstrated excellent internal consistency and strong convergent and discriminant validity.

Performance Anxiety. Performance anxiety and school-related anxiety were assessed using the School Anxiety subscale (15 items; score range 0–30) of the German revision of the Fear Survey Schedule for Children–Revised [15]. Items are rated on a 3-point Likert scale (0 = no fear at all, 1 = some fear, 2 = strong fear), with higher scores indicating greater anxiety severity. The German revision of the FSSC-R demonstrates good internal consistency (Cronbach's α typically ≥ .85 for the total scale, with acceptable-to-good reliability for individual subscales) and satisfactory convergent validity with other established anxiety measures.

Emotion regulation difficulties. Emotion regulation difficulties were assessed using the Difficulties in Emotion Regulation Scale – Short Form [16], an 18-item self-report measure derived from the original DERS. Items are rated on a 5-point Likert scale (1–5), yielding a total score range of 18–90, with higher scores indicating greater difficulties in emotion regulation. The DERS-SF comprises six subscales (Nonacceptance, Goals, Impulse, Awareness, Strategies, Clarity), each consisting of three items. It is a dimensional measure and no clinical cutoff score has been established. In validation studies, the scale demonstrated excellent internal consistency and strong convergent validity with related measures of emotional functioning.

Procrastination. Procrastination was assessed using the 4-item short version of the Procrastination Questionnaire for Students [17]; Items are rated on a 5-point Likert scale (1–5), yielding a total score range of 4–20, with higher scores indicating greater procrastination tendencies. The PFS-4 is a dimensional screening measure and no clinical cutoff score has been established. The original instrument demonstrated good internal consistency and satisfactory construct validity; short forms show comparable reliability in validation analyses.

Quality of life. Health-related quality of life was assessed using the KIDSCREEN-27 [18], a standardized self-report instrument for children and adolescents. The KIDSCREEN-27 comprises 27 items rated on a 5-point Likert scale, assessing frequency (e.g., never–always) or intensity (e.g., not at all–extremely) of experiences over the past week. Items are summed and transformed into T-scores (mean = 50, SD = 10) based on European normative data, with higher scores indicating better health-related quality of life. The instrument yields five subscales (Physical Well-Being, Psychological Well-Being, Autonomy & Parent Relations, Social Support & Peers, School Environment). The KIDSCREEN-27 is a dimensional measure; no clinical cutoff score is defined, and interpretation is based on normative comparisons. Validation studies across 13 European countries demonstrated good internal consistency across subscales and strong cross-cultural validity.

Wellbeing. Subjective well-being was assessed using the WHO-5 Well-Being Index [19], a brief self-report measure of positive psychological well-being. The WHO-5 consists of 5 items rated on a 6-point Likert scale (0–5) reflecting well-being during the past two weeks. Raw scores range from 0–25 and are multiplied by 4 to obtain a percentage score (0–100), with higher scores indicating better well-being. A cutoff score ≤50 on the transformed scale is commonly used to indicate reduced well-being and possible depression, while scores ≤28 suggest likely clinical depression and warrant further assessment. The WHO-5 is a unidimensional scale and validation studies demonstrate excellent internal consistency as well as strong construct validity and sensitivity to change in clinical and population samples.

Satisfaction (student and multiplier evaluation). Students's program satisfaction was assessed post-intervention using a single-item student evaluation. Participants rated the overall quality of the PROTECTtraining or PROTECTinfo on a 10-point scale (1 = very poor to 10 = very good), with higher scores indicating greater satisfaction. The measure captured participants' global subjective evaluation of the intervention. Additionally, the multipliers evaluated the intervention. Following intervention delivery, facilitators completed a structured post-intervention multiplier evaluation assessing program acceptability and feasibility. The evaluation included items on overall conviction,

© 2026 Lindenberg K et al. *JAMA Network Open.*

perceived preparedness, satisfaction with provided materials, and perceived competence during delivery, each rated on 4-point Likert scales. In addition, the multipliers' overall program satisfaction was assessed using a 10-point rating scale (1 = very poor to 10 = very good).

Intervention fidelity. Intervention fidelity was assessed after each session using standardized, module-specific supervisor rating forms for PROTECTtraining and PROTECTinfo. Independent supervisors documented session characteristics (e.g., duration, attendance, contextual factors) and evaluated manual adherence for each predefined intervention component. Items captured whether core elements were implemented as specified (e.g., correct execution of exercises, discussion of key contents, appropriate structuring of session phases). For PROTECTtraining, adherence was rated at the exercise level across core components including introduction, psychoeducation, metaphorical leading figures, cognitive restructuring, problem solving and behavioral activation, homework/conclusion, and—specifically in module 4—emotion-regulation strategies. For PROTECTinfo, adherence covered the respective media-literacy components (mobile phone use and privacy; real-world vs virtual communication; gaming and its risks and benefits; cyberbullying and digital rights), each combined with introduction and homework/conclusion elements. Adherence items were rated on a 3-point scale (0 = not adherent/goal not achieved/strong deviation from manual, 1 = partially adherent/goal partially achieved/minor deviations, 2 = completely adherent/goal achieved/manual-conform implementation). Global implementation quality and session management were additionally rated on a 6-point scale (1 = very good to 6 = inadequate). Module-level and overall adherence scores were derived from the item ratings. Supervisors also indicated whether they intervened during session delivery. Parallel forms with structurally equivalent scoring ensured comparability across intervention arms.

Harms. Harms related to unwanted effects of the interventions (i.e., symptom progression instead of symptom reduction) were assessed with the primary outcome measures by computing reliable deterioration analyses of the primary endpoint.

Additional measures.

Several additional constructs such as personality, the use of substances and less studied addictive behaviors, were assessed to explore associated risk factors and behaviors.

P**ersonality.** Personality traits were assessed at baseline using the Big Five Inventory for Children and Adolescents [20], a 30-item self-report measure assessing the five personality domains. Items are rated on a 5-point Likert scale (1 = does not apply at all to 5 = fully applies) with higher scores indicating stronger trait expression. The instrument shows good internal consistency and established factorial validity.

Problematic Alcohol Use. Alcohol use was assessed at baseline and at 12 months follow-up using the Alcohol Use Disorders Identification Test [21], a 10-item questionnaire with response options scored 0–4 depending on item format, yielding a total score range of 0–40. Scores ≥8 indicate hazardous use. The scale demonstrates high internal consistency and strong diagnostic validity.

Problematic Cannabis Use. Cannabis-related problems were measured at baseline and at 12 months follow-up with the Cannabis Abuse Screening Test [22], a 6-item self-report instrument rated on a 5-point frequency scale (0 = never to 4 = very often). Total scores range from 0–24, with scores ≥7 indicating risk of problematic use. Validation studies report good reliability.

Problematic Gambling. Gambling problems were screened at baseline and at 12 months follow-up using the Lie/Bet Questionnaire [23], a 2-item screening instrument with dichotomous responses (yes/no). Endorsement of ≥1 item indicates risk for pathological gambling. The tool shows high sensitivity and good specificity in adult populations.

Problematic Shopping. Compulsive buying symptoms were assessed at 12 months follow-up with the Pathological Buying Screener [24], a 13-item self-report scale rated on a 5-point Likert scale (1 = never to 5 = very frequently). Scores are summed, with higher values indicating greater symptom severity. The PBS demonstrates excellent internal consistency and strong convergent validity. Screening thresholds have been proposed but interpretation is generally dimensional. Problematic Shopping. Compulsive buying symptoms were assessed at t4 with the Pathological Buying Screener

(PBS; Müller et al., 2015), a 13-item self-report scale rated on a 5-point Likert scale (1 = never to 5 = very frequently). Scores are summed, with higher values indicating greater symptom severity. The PBS demonstrates excellent internal consistency and strong convergent validity. Validation analyses identified a cutoff score ≥29 as optimal for detecting probable pathological buying.

Problematic Pornography Use. Problematic pornography use was assessed at 12 months follow-up using the Problematic Pornography Consumption Scale – Short Form (PPCS-6; Bőthe et al., 2018), a 6-item measure rated on a 7-point Likert scale (1 = never to 7 = all the time). Higher total scores indicate greater symptom severity. research indicates a cutoff score of 20 or higher for identifying individuals at elevated risk of problematic use, although no universally established diagnostic threshold exists. The PPCS-6 shows excellent internal consistency and strong construct validity.

Sample Size

Sample size planning was informed by the prior efficacy trial of the PROTECT program (Lindenberg et al., 2022), which yielded small-to-moderate effects among at-risk adolescents. Sample size estimation was conducted using G*Power 3.1 based on a two-group comparison with a small standardized effect size (d = 0.20), an α level of .05, and a statistical power (1 − β) of .80. Under these assumptions, a minimum of 394 participants per group (N = 788 total) is required to detect a statistically significant between-group difference at the final follow-up.

The effect size was conservatively specified, as effectiveness trials conducted under real-world conditions typically yield smaller effects than efficacy trials conducted under highly controlled conditions (e.g., Becker & Stice, 2017). Accordingly, higher attrition rates were anticipated than in the preceding efficacy trial. Based on prior implementation studies, approximately 30% attrition was expected at each follow-up assessment (4 and 12 months).

Starting from an initial target sample of 1,800 participants, this projection yields approximately 1,200 participants at 4-month follow-up and approximately 880 participants at 12-month follow-up. Even allowing for unequal dropout across conditions, this sample size was deemed sufficient to retain at least 394 participants per group at the final assessment, thereby preserving adequate statistical power for primary between-group comparisons. These assumptions were intentionally conservative with respect to both effect size and attrition, reflecting ethical considerations that the consequences of an underpowered effectiveness trial (i.e., inconclusive results in a large-scale public health intervention) would outweigh those of recruiting a larger-than-necessary initial sample. The planned baseline sample also ensured sufficient power for differential analyses across specific forms of internet use (gaming, social media).

Statistical Methods

Outcome Comparison. All data analyses were performed using R version 4.5.1. All statistical tests were two-sided, with a significance threshold set at $P < .05$. Descriptive statistics for pretest data were computed for observed data.

To evaluate differences in primary and secondary endpoints, three-level hierarchical linear models (HLMs) were employed to account for the nested structure of the repeated-measures design (level 1: time, level 2: participants, level 3: classes), using the R package lme4 version 1.1–37. Consistent with the recommendations of Coffman et al.[25], constrained longitudinal data analyses (cLDA) were performed using HLMs including time and group × time interaction as fixed effects. cLDA constrains pretest means to be equal between groups. HLMs included random intercepts at levels 2 and 3. Time was treated as a categorical variable using dummy coding, with pretest (T1) as the reference category and separate indicators for the 1-month (T2), 4-months (T3), and 12-months follow-up assessments (T4), accounting for discontinuous change over time. Group was binary coded (1 = PROTECTtraining intervention, 0 = PROTECTinfo control group). Thus, fixed effects estimates for time represent changes in the outcome relative to pretest values within the control group. Because pretest means were constrained to be equal across groups, the group × time interaction coefficients represent both the differential change from pretest and the between-group difference at the corresponding follow-up assessment. HLMs were specified as follow:

$$\text{Outcome} \sim \text{Time}_{T2vsT1} + \text{Time}_{T3vsT1} + \text{Time}_{T4vsT1} + \text{Group}\times\text{Time}_{T2vsT1} + \text{Group}\times\text{Time}_{T3vsT1} + \text{Group}\times\text{Time}_{T4vsT1} + (1 \mid \text{Class} / \text{ID})$$

© 2026 Lindenberg K et al. *JAMA Network Open.*

Between-group effect sizes were calculated as Cohen's *d*, using estimated mean differences and pooled observed standard deviations (*SD*s) at the respective time point. Within-group effect sizes were calculated as the estimated mean difference between pretest and 12-month follow-up divided by the pooled observed SD at 12-month follow-up[26].

Responder Analysis. In participants who provided complete data at 12-month follow-up, responder status was determined using the Reliable Change Index (RCI[27]), which identifies whether an individual's change exceeds the amount expected due to measurement error alone. Participants were classified as reliably changed when the absolute RCI exceeded 1.96, corresponding to a statistically significant individual change. Using the observed pretest *SD* and the internal consistency of the primary endpoint as estimated by McDonald's ω_{within}, a reduction or increase of 9.36 points in the CSAS from pretest to 12-months follow-up was classified as reliably improved (i.e., response) or deteriorated, respectively. Frequencies of reliable change between groups were compared using chi-square tests.

Analysis Population. Analyses of primary and secondary endpoints were conducted according to the intention-to-treat (ITT) principle, including all randomized participants (see CONSORT flow diagram). Analyses were repeated for subgroups classified as 'high-risk' (n = 205) and 'not at risk' (n = 1,588) according to the number of fulfilled diagnostic criteria for Internet Gaming Disorder and Unspecified Internet Use Disorder at pretest as defined by the primary endpoint (i.e., ≥2 out of 9 criteria in ≥50% of imputed datasets). Due to convergence issues in the three-level models arising from small sample sizes in the 'high-risk' subgroup, analyses for this subgroup were conducted using two-level models with observations nested within participants, omitting 'class' as the third level.

Sensitivity Analyses.

Missing Data. Missing data were addressed using multiple imputation by chained equations (MICE), implemented with the R package mice version 3.18.0 (van Buuren & Groothuis-Oudshoorn, 2011). For the primary endpoint (CSAS), imputation was performed at the item level prior to the calculation of total scores (Gottschall et al., 2012). For secondary endpoints, imputation was performed at the scale level to reduce computational burden.

To ensure the use of all informative variables in imputing missing values, the predictor matrix was generated based on correlations and usable cases using the 'quickpred' function within mice, with minimum thresholds set at r = .05 and ≥5% usable cases. Imputations were generated separately for the intervention and control group (groupwise imputation) using the 'bygroup' function within miceadds[28], and retrospective prediction was excluded. Fifty imputed datasets were generated. Analyses were conducted separately for each imputed dataset, with results pooled according to Rubin's rules (Rubin, 1987).

Additional Analyses. Furthermore, analyses of the primary endpoint were repeated within a completer subsample (n = 794) that provided complete datasets at all time points and within a per-protocol subsample (n = 567) that attended all four sessions and provided complete datasets at all time points.

© 2026 Lindenberg K et al. *JAMA Network Open.*

eTable 1. Measures and Assessment Time Points

Measures	Pretest	Posttest	4 months	12 months
Sociodemographics and internet usage				
Age, gender,acacemic level	x	-	-	-
Academic performance	x	-	-	-
Time spent online (gaming)				
Time spent online (social media)				
Primary outcome				
Modified CSAS	x	x	x	x
Secondary outcome				
Short CIUS	x	x	x	x
IGD-9	x	x	x	x
GDT	x	x	x	x
GAT	x	x	x	x
SMDS	x	x	x	x
SDQ	x	x	x	x
Conners-3 Index	x	x	x	x
DesTeen	x	x	x	x
SIAS	x	x	x	x
FSSC-R Performance Anxiety	x	x	x	x
DERS-SF	x	x	x	x
PFS-4	x	x	x	x
KIDSSCREEN-27	x	x	x	x
WHO-5	x	x	x	x
Satisfaction (Student Evaluation)	-	x	-	-
Satisfaction (Multiplier Evaluation)	-	x	-	-
Intervention Fidelity (weekly)	-	x	-	-
Harms	x	x	x	x
Other measures				
BFI-K KJ	x	-	-	-
Lie/bet Questionnaire	x	-	-	x
AUDIT	x	-	-	x
CAST	x	-	-	x
PBS	-	-	-	x
PPCS-6	-	-	-	x

Note. Modified CSAS, modified version of the video game dependency scale to capture Gaming and unspecified Internet use disorders, short CIUS, short Compulsive Internet Use Scale, IGD-9, Internet Gaming Disorder Scale; GDT, Gaming Disorder Test; GAT, Gaming Attitude Test; SMDS, Social Media Disorder Scale; SDQ, Strength and Difficulties Questionnaire; Conners-3 Index, Conners 3rd Edition ADHD Index; DesTeen, Depression Screener for Teenagers; SIAS, Social Interaction Anxiety Scale; FSSC-R, Fear Survey Schedule for Children – Revised; DERS-SF, Difficulties in Emotion Regulation Scale – Short Form; PFS-4, 4-item version of the Procrastination Questionnaire for Students; KIDSCREEN-27, 27-item version of the KIDSCREEN questionnaire; WHO-5, WHO Well-Being Index; BFI-K/KJ, Big Five Inventory for Children and Adolescents; Lie/Bet Questionnaire, screening instrument for pathological gambling; AUDIT, Alcohol Use Disorders Identification Test; CAST, Cannabis Abuse Screening Test; PBS, Pathological Buying Screener; PPCS-6, 6-item version of the Problematic Pornography Consumption Scale.

© 2026 Lindenberg K et al. *JAMA Network Open.*

eTable 2. Completer Analysis. Estimated Marginal Means, Observed *SD*s, and Effect Sizes of the Primary Outcome

	Pretest	Posttest	4 months	12 months	d_{within}, 95% CI (Pretest vs 12 months)
	M(*SD*)	*M*(*SD*)	*M*(*SD*)	*M*(*SD*)	
Total sample (*n*=794)[1]					
$PROTECT_{training}$	8.54 (7.50)	7.94 (8.16)	7.11 (7.94)	7.40 (7.49)	-0.15 [-0.23, -0.07]
$PROTECT_{info}$	8.54 (7.94)	7.47 (7.62)	6.99 (7.77)	7.59 (7.92)	-0.12 [-0.21, -0.04]
$d_{between}$, 95% CI	-	0.06 [-0.05, 0.17]	0.02 [-0.09, 0.12]	-0.02 [-0.13, 0.09]	
P value	-	.28	.78	.67	
High-risk (*n*=74)[2]					
$PROTECT_{training}$	22.81 (8.62)	18.97 (11.65)	15.67 (10.88)	13.39 (8.97)	-0.99 [-1.31, -0.66]
$PROTECT_{info}$	22.81 (8.22)	16.87 (9.80)	15.34 (11.08)	15.31 (10.09)	-0.78 [-1.10, -0.47]
$d_{between}$, 95% CI	-	0.20 [-0.18, 0.57]	0.03 [-0.33, 0.39]	-0.20 [-0.62, 0.22]	
P value	-	.30	.87	.35	
Not at risk (*n*=720)[1]					
$PROTECT_{training}$	7.05 (5.85)	6.76 (6.87)	6.19 (7.09)	6.73 (7.09)	-0.04 [-0.13, 0.04]
$PROTECT_{info}$	7.05 (6.02)	6.49 (6.57)	6.12 (6.72)	6.79 (7.15)	-0.04 [-0.12, 0.05]
$d_{between}$, 95% CI	-	0.04 [-0.08, 0.16]	0.01 [-0.11, 0.13]	-0.01 [-0.12, 0.10]	
P value	-	.50	.87	.89	

Note. [1]3-level model (observations in participants in classes), [2]2-level model (observations in participants). Estimated marginal means were derived from hierarchical linear models including time and the group × time interaction as fixed effects. Time was treated as a factor (0, 1, 4, and 12 months). Primary outcome is the modified version of the video game dependency scale at 12 months to capture Gaming and unspecified Internet use disorders.

© 2026 Lindenberg K et al. *JAMA Network Open.*

eTable 3. Per-Protocol Analysis. Estimated Marginal Means, Observed *SD*s, and Effect Sizes of the Primary Outcome

	Pretest	Posttest	4 months	12 months	d_{within}, 95% CI (Pretest vs 12 months)
	M(*SD*)	*M*(*SD*)	*M*(*SD*)	*M*(*SD*)	
Total sample (*n*=567)[1]					
$PROTECT_{training}$	8.71 (8.03)	8.07 (8.55)	7.17 (8.26)	7.24 (7.60)	-0.19 [-0.29, -0.09]
$PROTECT_{info}$	8.71 (7.41)	7.37 (6.96)	6.68 (6.77)	7.57 (7.55)	-0.15 [-0.25, -0.05]
$d_{between}$, 95% CI	-	0.09 [-0.04, 0.22]	0.06 [-0.07, 0.20]	-0.04 [-0.18, 0.09]	
P value	-	.16	.34	.51	
High-risk (*n*=51)[2]					
$PROTECT_{training}$	22.78 (9.16)	19.01 (12.20)	14.98 (11.46)	12.58 (9.23)	-1.09 [-1.43, -0.76]
$PROTECT_{info}$	22.78 (7.81)	13.89 (7.54)	11.08 (7.28)	14.94 (9.48)	-0.84 [-1.23, -0.45]
$d_{between}$, 95% CI	-	0.49 [0.06, 0.91]	0.39 [-0.06, 0.84]	-0.25 [-0.73, 0.22]	
P value	-	.03	.09	.30	
Not at risk (*n*=516)[1]					
$PROTECT_{training}$	7.32 (6.15)	6.93 (7.00)	6.40 (7.30)	6.78 (7.16)	-0.08 [-0.18, 0.03]
$PROTECT_{info}$	7.32 (5.96)	6.69 (6.62)	6.17 (6.59)	6.83 (7.04)	-0.07 [-0.17, 0.03]
$d_{between}$, 95% CI	-	0.04 [-0.10, 0.18]	0.03 [-0.10, 0.17]	-0.01 [-0.14, 0.13]	
P value	-	.61	.62	.92	

Note. [1]3-level model (observations in participants in classes), [2]2-level model (observations in participants). Estimated marginal means were derived from hierarchical linear models including time and the group × time interaction as fixed effects. Time was treated as a factor (0, 1, 4, and 12 months). Primary outcome is the modified version of the video game dependency scale at 12 months to capture Gaming and unspecified Internet use disorders.

© 2026 Lindenberg K et al. *JAMA Network Open.*

eTable 4. Secondary Outcomes. Estimated Marginal Means, Observed *SD*s, and Effect Sizes of Secondary Outcomes After Multiple Imputation (Intention-to-Treat Analyses)

	Pretest	Posttest	4 months	12 months	d_{within}, 95% *CI* (Pretest vs 12 months)
	M(*SD*)	*M*(*SD*)	*M*(*SD*)	*M*(*SD*)	
Compulsive internet use, CIUS					
Total sample (*N*=1793)[1]					
$PROTECT_{training}$	6.72 (4.22)	6.38 (4.07)	5.81 (4.12)	6.55 (4.21)	-0.04 [-0.10, 0.02]
$PROTECT_{info}$	6.72 (4.24)	6.13 (3.99)	5.94 (4.11)	6.67 (4.30)	-0.01 [-0.08, 0.06]
$d_{between}$, 95% *CI*	-	0.06 [-0.03, 0.15]	-0.03 [-0.12, 0.05]	-0.03 [-0.11, 0.06]	
P value	-	.17	.46	.54	
High-risk (*n*=205)[2]					
$PROTECT_{training}$	11.68 (4.20)	10.21 (4.37)	8.94 (4.54)	9.60 (4.55)	-0.44 [-0.63, -0.25]
$PROTECT_{info}$	11.68 (4.29)	9.64 (4.28)	9.25 (4.45)	9.83 (4.89)	-0.39 [-0.59, -0.19]
$d_{between}$, 95% *CI*	-	0.13 [-0.14, 0.40]	-0.07 [-0.33, 0.19]	-0.05 [-0.30, 0.20]	
P value	-	.34	.61	.71	
Not at risk (*n*=1588)[1]					
$PROTECT_{training}$	6.08 (3.80)	5.88 (3.76)	5.40 (3.89)	6.16 (4.01)	0.02 [-0.05, 0.09]
$PROTECT_{info}$	6.08 (3.75)	5.67 (3.71)	5.51 (3.86)	6.25 (4.03)	0.04 [-0.03, 0.12]
$d_{between}$, 95% *CI*	-	0.06 [-0.04, 0.15]	-0.03 [-0.12, 0.07]	-0.02 [-0.12, 0.07]	
P value	-	.24	.57	.62	
Gaming attitudes, preoccupation, GAT					
Total sample (*N*=1793)[1]					
$PROTECT_{training}$	27.61 (13.52)	25.79 (13.41)	25.37 (13.41)	25.53 (13.73)	-0.15 [-0.22, -0.09]
$PROTECT_{info}$	27.61 (13.50)	25.32 (13.81)	25.02 (13.71)	24.91 (13.41)	-0.20 [-0.27, -0.13]
$d_{between}$, 95% *CI*	-	0.03 [-0.05, 0.11]	0.03 [-0.06, 0.11]	0.05 [-0.04, 0.14]	
P value	-	.40	.55	.32	
High-risk (*n*=205)[2]					
$PROTECT_{training}$	40.23 (16.65)	36.15 (16.61)	34.34 (15.94)	34.46 (16.30)	-0.36 [-0.55, -0.17]
$PROTECT_{info}$	40.23 (16.10)	36.94 (18.67)	34.21 (16.61)	31.41 (15.86)	-0.55 [-0.73, -0.36]
$d_{between}$, 95% *CI*	-	-0.05 [-0.26, 0.17]	0.01 [-0.24, 0.26]	0.19 [-0.06, 0.44]	
P value	-	.68	.95	.14	
Not at risk (*n*=1588)[1]					
$PROTECT_{training}$	25.99 (12.29)	24.45 (12.40)	24.21 (12.66)	24.38 (12.98)	-0.12 [-0.19, -0.06]
$PROTECT_{info}$	25.99 (12.05)	23.84 (12.21)	23.85 (12.76)	24.08 (12.78)	-0.15 [-0.22, -0.07]
$d_{between}$, 95% *CI*	-	0.05 [-0.04, 0.14]	0.03 [-0.06, 0.12]	0.02 [-0.07, 0.12]	
P value	-	.28	.53	.62	
Gaming attitudes, trivializing, GAT					
Total sample (*N*=1793)[1]					
$PROTECT_{training}$	36.33 (14.42)	37.20 (15.62)	39.40 (15.83)	38.97 (14.26)	0.19 [0.11, 0.27]
$PROTECT_{info}$	36.33 (14.12)	36.56 (15.22)	38.30 (15.91)	38.32 (13.53)	0.14 [0.07, 0.22]
$d_{between}$, 95% *CI*	-	0.04 [-0.04, 0.13]	0.07 [-0.02, 0.16]	0.05 [-0.05, 0.15]	
P value	-	.35	.14	.37	
High-risk (*n*=205)[2]					
$PROTECT_{training}$	35.88 (13.33)	36.52 (15.00)	39.06 (15.06)	39.26 (15.36)	0.24 [0.01, 0.46]
$PROTECT_{info}$	35.88 (14.45)	34.70 (13.59)	39.83 (15.49)	38.05 (12.85)	0.15 [-0.07, 0.38]
$d_{between}$, 95% *CI*	-	0.13 [-0.16, 0.41]	-0.05 [-0.33, 0.23]	0.09 [-0.21, 0.38]	
P value	-	.38	.73	.57	
Not at risk (*n*=1588)[1]					
$PROTECT_{training}$	36.41 (14.57)	37.30 (15.70)	39.45 (15.93)	38.94 (14.11)	0.18 [0.10, 0.27]
$PROTECT_{info}$	36.41 (14.08)	36.82 (15.40)	38.12 (15.96)	38.38 (13.61)	0.14 [0.06, 0.22]
$d_{between}$, 95% *CI*	-	0.03 [-0.06, 0.12]	0.08 [-0.01, 0.18]	0.04 [-0.07, 0.15]	
P value	-	.50	.09	.46	

(*To be continued*)

© 2026 Lindenberg K et al. *JAMA Network Open.*

eTable 4. (*Continued*)

	Pretest	Posttest	4 months	12 months	d_{within}, 95% *CI* (Pretest vs 12 months)
	M(*SD*)	*M*(*SD*)	*M*(*SD*)	*M*(*SD*)	
IGD symptoms, GDT					
Total sample (*N*=1793)[1]					
$PROTECT_{training}$	6.67 (2.62)	6.48 (2.69)	6.24 (2.65)	6.19 (2.65)	-0.18 [-0.26, -0.11]
$PROTECT_{info}$	6.67 (2.88)	6.43 (2.75)	6.27 (2.89)	6.19 (2.70)	-0.18 [-0.26, -0.10]
$d_{between}$, 95% *CI*	-	0.02 [-0.07, 0.11]	-0.01 [-0.10, 0.08]	-0.00 [-0.11, 0.10]	
P value	-	.66	.84	.96	
High-risk (*n*=205)[2]					
$PROTECT_{training}$	9.23 (3.41)	8.59 (3.53)	8.38 (3.54)	7.53 (3.23)	-0.51 [-0.74, -0.28]
$PROTECT_{info}$	9.23 (3.83)	8.81 (3.70)	8.97 (4.32)	7.75 (3.44)	-0.44 [-0.68, -0.21]
$d_{between}$, 95% *CI*	-	-0.06 [-0.33, 0.20]	-0.15 [-0.40, 0.11]	-0.06 [-0.37, 0.24]	
P value	-	.65	.26	.67	
Not at risk (*n*=1588)[1]					
$PROTECT_{training}$	6.34 (2.39)	6.21 (2.46)	5.96 (2.40)	6.01 (2.53)	-0.13 [-0.21, -0.06]
$PROTECT_{info}$	6.34 (2.46)	6.13 (2.40)	5.93 (2.40)	6.00 (2.49)	-0.14 [-0.22, -0.05]
$d_{between}$, 95% *CI*	-	0.03 [-0.07, 0.13]	0.01 [-0.09, 0.12]	0.01 [-0.10, 0.11]	
P value	-	.52	.78	.93	
IGD symptoms, IGD-9					
Total sample (*N*=1793)[1]					
$PROTECT_{training}$	1.84 (1.84)	1.72 (1.96)	1.61 (1.93)	1.58 (1.81)	-0.14 [-0.21, -0.07]
$PROTECT_{info}$	1.84 (1.79)	1.74 (1.92)	1.64 (1.95)	1.67 (1.86)	-0.10 [-0.17, -0.02]
$d_{between}$, 95% *CI*	-	-0.01 [-0.10, 0.07]	-0.02 [-0.10, 0.07]	-0.05 [-0.14, 0.05]	
P value	-	.74	.72	.34	
High-risk (*n*=205)[2]					
$PROTECT_{training}$	3.67 (2.09)	3.45 (2.54)	3.01 (2.49)	2.84 (2.30)	-0.36 [-0.57, -0.15]
$PROTECT_{info}$	3.67 (2.15)	3.56 (2.40)	3.24 (2.42)	2.73 (2.29)	-0.41 [-0.62, -0.20]
$d_{between}$, 95% *CI*	-	-0.04 [-0.29, 0.21]	-0.09 [-0.34, 0.16]	0.05 [-0.23, 0.33]	
P value	-	.73	.47	.74	
Not at risk (*n*=1588)[1]					
$PROTECT_{training}$	1.61 (1.68)	1.49 (1.76)	1.43 (1.76)	1.42 (1.67)	-0.11 [-0.18, -0.04]
$PROTECT_{info}$	1.61 (1.59)	1.51 (1.71)	1.44 (1.77)	1.53 (1.75)	-0.05 [-0.13, 0.04]
$d_{between}$, 95% *CI*	-	-0.01 [-0.11, 0.08]	-0.00 [-0.10, 0.09]	-0.06 [-0.17, 0.04]	
P value	-	.81	.94	.23	
SMD symptoms, SMDS					
Total sample (*N*=1793)[1]					
$PROTECT_{training}$	1.60 (1.90)	1.56 (1.94)	1.48 (1.89)	1.62 (1.97)	0.01 [-0.06, 0.08]
$PROTECT_{info}$	1.60 (1.90)	1.57 (2.02)	1.47 (1.89)	1.63 (1.91)	0.01 [-0.06, 0.09]
$d_{between}$, 95% *CI*	-	-0.01 [-0.09, 0.07]	0.01 [-0.09, 0.10]	-0.01 [-0.10, 0.09]	
P value	-	.83	.90	.90	
High-risk (*n*=205)[2]					
$PROTECT_{training}$	3.65 (2.39)	3.56 (2.76)	2.99 (2.45)	3.02 (2.60)	-0.25 [-0.44, -0.06]
$PROTECT_{info}$	3.65 (2.45)	3.23 (2.70)	3.01 (2.47)	3.01 (2.47)	-0.25 [-0.46, -0.04]
$d_{between}$, 95% *CI*	-	0.12 [-0.12, 0.36]	-0.01 [-0.28, 0.27]	0.00 [-0.26, 0.27]	
P value	-	.32	.95	.98	
Not at risk (*n*=1588)[1]					
$PROTECT_{training}$	1.33 (1.66)	1.29 (1.64)	1.28 (1.71)	1.43 (1.79)	0.05 [-0.02, 0.13]
$PROTECT_{info}$	1.33 (1.63)	1.35 (1.80)	1.26 (1.69)	1.44 (1.75)	0.06 [-0.02, 0.14]
$d_{between}$, 95% *CI*	-	-0.03 [-0.13, 0.06]	0.01 [-0.09, 0.11]	-0.01 [-0.11, 0.10]	
P value	-	.46	.82	.92	

(*To be continued*)

© 2026 Lindenberg K et al. *JAMA Network Open.*

eTable 4. (*Continued*)

	Pretest	Posttest	4 months	12 months	d_{within}, 95% *CI* (Pretest vs 12 months)
	M(*SD*)	*M*(*SD*)	*M*(*SD*)	*M*(*SD*)	
Daily time spent gaming, hours					
Total sample (*N*=1793)[1]					
$PROTECT_{training}$	2.20 (1.89)	2.19 (1.99)	2.19 (2.10)	2.14 (2.07)	-0.03 [-0.12, 0.06]
$PROTECT_{info}$	2.20 (1.99)	2.15 (2.05)	2.06 (2.10)	2.05 (1.91)	-0.08 [-0.15, -0.00]
$d_{between}$, 95% *CI*	-	0.02 [-0.07, 0.11]	0.06 [-0.03, 0.15]	0.05 [-0.06, 0.15]	
P value	-	.64	.18	.38	
High-risk (*n*=205)[2]					
$PROTECT_{training}$	3.34 (2.49)	3.13 (2.41)	2.96 (2.52)	3.06 (2.53)	-0.12 [-0.35, 0.11]
$PROTECT_{info}$	3.34 (2.58)	3.47 (2.88)	3.27 (2.85)	2.83 (2.38)	-0.21 [-0.43, 0.01]
$d_{between}$, 95% *CI*	-	-0.13 [-0.38, 0.12]	-0.12 [-0.40, 0.16]	0.09 [-0.20, 0.38]	
P value	-	.32	.41	.53	
Not at risk (*n*=1588)[1]					
$PROTECT_{training}$	2.05 (1.75)	2.06 (1.91)	2.08 (2.02)	2.02 (1.98)	-0.02 [-0.10, 0.07]
$PROTECT_{info}$	2.05 (1.84)	1.98 (1.84)	1.91 (1.92)	1.95 (1.82)	-0.05 [-0.13, 0.02]
$d_{between}$, 95% *CI*	-	0.04 [-0.05, 0.14]	0.09 [-0.00, 0.19]	0.04 [-0.07, 0.15]	
P value	-	.35	.06	.51	
Daily time spent on social media, hours					
Total sample (*N*=1793)[1]					
$PROTECT_{training}$	2.82 (2.36)	2.83 (2.31)	3.09 (2.35)	3.20 (2.27)	0.17 [0.10, 0.24]
$PROTECT_{info}$	2.82 (2.34)	2.77 (2.32)	3.01 (2.34)	3.17 (2.33)	0.15 [0.08, 0.22]
$d_{between}$, 95% *CI*	-	0.03 [-0.06, 0.11]	0.03 [-0.06, 0.12]	0.01 [-0.08, 0.10]	
P value	-	.54	.49	.76	
High-risk (*n*=205)[2]					
$PROTECT_{training}$	4.17 (2.89)	3.96 (2.88)	4.10 (2.60)	3.97 (2.49)	-0.08 [-0.28, 0.13]
$PROTECT_{info}$	4.17 (2.87)	4.11 (2.95)	4.29 (2.85)	4.54 (2.94)	0.13 [-0.09, 0.35]
$d_{between}$, 95% *CI*	-	-0.05 [-0.28, 0.18]	-0.07 [-0.35, 0.21]	-0.21 [-0.49, 0.07]	
P value	-	.67	.62	.14	
Not at risk (*n*=1588)[1]					
$PROTECT_{training}$	2.65 (2.20)	2.68 (2.17)	2.96 (2.27)	3.11 (2.21)	0.21 [0.14, 0.29]
$PROTECT_{info}$	2.65 (2.20)	2.60 (2.17)	2.85 (2.21)	3.00 (2.17)	0.16 [0.09, 0.24]
$d_{between}$, 95% *CI*	-	0.04 [-0.05, 0.13]	0.05 [-0.04, 0.14]	0.05 [-0.05, 0.15]	
P value	-	.41	.32	.32	
Internalizing psychopathology, SDQ					
Total sample (*N*=1793)[1]					
$PROTECT_{training}$	6.30 (3.63)	6.37 (3.86)	6.44 (3.86)	6.40 (3.87)	0.03 [-0.04, 0.09]
$PROTECT_{info}$	6.30 (3.75)	6.11 (3.82)	6.53 (4.12)	6.67 (4.17)	0.09 [0.01, 0.18]
$d_{between}$, 95% *CI*	-	0.07 [-0.02, 0.15]	-0.02 [-0.11, 0.06]	-0.07 [-0.16, 0.03]	
P value	-	.14	.60	.17	
High-risk (*n*=205)[2]					
$PROTECT_{training}$	8.54 (4.05)	8.77 (4.22)	8.41 (4.06)	7.71 (3.90)	-0.20 [-0.40, 0.00]
$PROTECT_{info}$	8.54 (4.03)	8.40 (4.02)	8.33 (4.10)	8.75 (4.55)	0.05 [-0.17, 0.27]
$d_{between}$, 95% *CI*	-	0.09 [-0.17, 0.35]	0.02 [-0.26, 0.30]	-0.25 [-0.53, 0.03]	
P value	-	.49	.90	.09	
Not at risk (*n*=1588)[1]					
$PROTECT_{training}$	6.01 (3.44)	6.06 (3.68)	6.19 (3.75)	6.24 (3.83)	0.06 [-0.02, 0.13]
$PROTECT_{info}$	6.01 (3.62)	5.82 (3.69)	6.30 (4.06)	6.40 (4.04)	0.10 [0.01, 0.19]
$d_{between}$, 95% *CI*	-	0.07 [-0.03, 0.16]	-0.03 [-0.12, 0.06]	-0.04 [-0.14, 0.06]	
P value	-	.18	.55	.43	

(*To be continued*)

eTable 4. (*Continued*)

	Pretest	Posttest	4 months	12 months	d_{within}, 95% *CI* (Pretest vs 12 months)
	M(*SD*)	*M*(*SD*)	*M*(*SD*)	*M*(*SD*)	
Externalizing psychopathology, SDQ					
Total sample (*N*=1793)[1]					
$PROTECT_{training}$	6.02 (3.47)	5.87 (3.51)	6.15 (3.74)	6.06 (3.70)	0.01 [-0.06, 0.08]
$PROTECT_{info}$	6.02 (3.37)	5.91 (3.52)	5.96 (3.60)	6.21 (3.69)	0.05 [-0.03, 0.13]
$d_{between}$, 95% *CI*	-	-0.01 [-0.10, 0.08]	0.05 [-0.04, 0.15]	-0.04 [-0.14, 0.06]	
P value	-	.80	.26	.42	
High-risk (*n*=205)[2]					
$PROTECT_{training}$	8.44 (3.54)	7.94 (3.67)	7.90 (3.40)	7.73 (3.72)	-0.19 [-0.41, 0.03]
$PROTECT_{info}$	8.44 (3.58)	8.40 (3.69)	7.95 (3.77)	8.04 (3.83)	-0.11 [-0.33, 0.12]
$d_{between}$, 95% *CI*	-	-0.12 [-0.40, 0.15]	-0.01 [-0.30, 0.27]	-0.08 [-0.37, 0.20]	
P value	-	.37	.93	.56	
Not at risk (*n*=1588)[1]					
$PROTECT_{training}$	5.71 (3.32)	5.60 (3.38)	5.93 (3.71)	5.84 (3.63)	0.04 [-0.04, 0.11]
$PROTECT_{info}$	5.71 (3.22)	5.59 (3.37)	5.70 (3.50)	5.97 (3.61)	0.07 [-0.01, 0.16]
$d_{between}$, 95% *CI*	-	0.00 [-0.09, 0.10]	0.06 [-0.03, 0.16]	-0.04 [-0.14, 0.07]	
P value	-	.96	.21	.50	
HrQoL, KIDSCREEN-27					
Total sample (*N*=1793)[1]					
$PROTECT_{training}$	105.48 (15.50)	104.83 (16.77)	103.19 (17.94)	102.48 (17.17)	-0.18 [-0.24, -0.11]
$PROTECT_{info}$	105.48 (15.71)	105.24 (17.09)	103.27 (19.32)	102.65 (16.82)	-0.17 [-0.23, -0.10]
$d_{between}$, 95% *CI*	-	-0.02 [-0.10, 0.06]	-0.00 [-0.08, 0.07]	-0.01 [-0.10, 0.08]	
P value	-	.56	.92	.83	
High-risk (*n*=205)[2]					
$PROTECT_{training}$	93.43 (17.65)	93.70 (19.47)	92.64 (18.43)	93.72 (19.33)	0.01 [-0.17, 0.20]
$PROTECT_{info}$	93.43 (17.92)	92.44 (20.03)	88.55 (22.67)	92.44 (19.72)	-0.05 [-0.24, 0.13]
$d_{between}$, 95% *CI*	-	0.06 [-0.17, 0.30]	0.20 [-0.04, 0.44]	0.07 [-0.18, 0.31]	
P value	-	.60	.10	.61	
Not at risk (*n*=1588)[1]					
$PROTECT_{training}$	107.05 (14.29)	106.28 (15.71)	104.57 (17.31)	103.62 (16.45)	-0.21 [-0.28, -0.14]
$PROTECT_{info}$	107.05 (14.78)	106.91 (15.99)	105.18 (18.03)	104.00 (15.97)	-0.19 [-0.26, -0.11]
$d_{between}$, 95% *CI*	-	-0.04 [-0.13, 0.05]	-0.03 [-0.12, 0.05]	-0.02 [-0.12, 0.07]	
P value	-	.38	.41	.64	
Well-being, WHO-5					
Total sample (*N*=1793)[1]					
$PROTECT_{training}$	15.10 (5.65)	15.51 (6.09)	14.98 (6.48)	14.65 (6.01)	-0.07 [-0.15, -0.00]
$PROTECT_{info}$	15.10 (5.52)	15.23 (6.04)	14.96 (6.64)	14.42 (6.17)	-0.11 [-0.19, -0.04]
$d_{between}$, 95% *CI*	-	0.05 [-0.04, 0.14]	0.00 [-0.08, 0.09]	0.04 [-0.06, 0.13]	
P value	-	.30	.95	.44	
High-risk (*n*=205)[2]					
$PROTECT_{training}$	11.91 (6.23)	12.33 (6.83)	11.33 (6.49)	12.06 (6.00)	0.02 [-0.19, 0.23]
$PROTECT_{info}$	11.91 (5.90)	12.49 (6.34)	11.66 (7.12)	12.14 (6.83)	0.04 [-0.18, 0.25]
$d_{between}$, 95% *CI*	-	-0.03 [-0.29, 0.24]	-0.05 [-0.31, 0.22]	-0.01 [-0.29, 0.26]	
P value	-	.85	.72	.93	
Not at risk (*n*=1588)[1]					
$PROTECT_{training}$	15.51 (5.41)	15.92 (5.85)	15.45 (6.32)	14.98 (5.92)	-0.09 [-0.17, -0.01]
$PROTECT_{info}$	15.51 (5.35)	15.58 (5.91)	15.39 (6.46)	14.72 (6.02)	-0.13 [-0.21, -0.06]
$d_{between}$, 95% *CI*	-	0.06 [-0.04, 0.15]	0.01 [-0.08, 0.10]	0.04 [-0.06, 0.14]	
P value	-	.24	.84	.39	

(*To be continued*)

eTable 4. (*Continued*)

	Pretest	Posttest	4 months	12 months	d_{within}, 95% *CI* (Pretest vs 12 months)
	M(*SD*)	*M*(*SD*)	*M*(*SD*)	*M*(*SD*)	
Depressive symptoms, DesTeen					
Total sample (*N*=1793)[1]					
PROTECT$_{training}$	11.34 (7.02)	11.13 (7.53)	11.65 (7.73)	11.60 (7.37)	0.03 [-0.03, 0.10]
PROTECT$_{info}$	11.34 (7.43)	10.78 (7.34)	11.39 (7.79)	11.89 (7.93)	0.07 [0.00, 0.14]
$d_{between}$, 95% *CI*	-	0.05 [-0.04, 0.13]	0.03 [-0.05, 0.11]	-0.04 [-0.13, 0.05]	
P value	-	.27	.42	.40	
High-risk (*n*=205)[2]					
PROTECT$_{training}$	17.11 (7.84)	16.05 (8.88)	16.63 (8.72)	16.36 (7.75)	-0.09 [-0.28, 0.10]
PROTECT$_{info}$	17.11 (8.17)	16.30 (7.98)	16.27 (8.27)	16.19 (8.94)	-0.11 [-0.31, 0.09]
$d_{between}$, 95% *CI*	-	-0.03 [-0.27, 0.21]	0.04 [-0.20, 0.28]	0.02 [-0.24, 0.28]	
P value	-	.82	.73	.87	
Not at risk (*n*=1588)[1]					
PROTECT$_{training}$	10.60 (6.53)	10.49 (7.08)	11.00 (7.33)	10.98 (7.08)	0.05 [-0.02, 0.12]
PROTECT$_{info}$	10.60 (6.96)	10.08 (6.91)	10.78 (7.47)	11.35 (7.60)	0.10 [0.03, 0.17]
$d_{between}$, 95% *CI*	-	0.06 [-0.03, 0.15]	0.03 [-0.05, 0.12]	-0.05 [-0.14, 0.04]	
P value	-	.21	.48	.30	
Social anxiety symptoms, SIAS					
Total sample (*N*=1793)[1]					
PROTECT$_{training}$	23.54 (13.63)	23.60 (14.12)	23.63 (14.72)	24.13 (14.51)	0.04 [-0.03, 0.11]
PROTECT$_{info}$	23.54 (13.99)	22.58 (13.79)	22.83 (13.77)	24.52 (14.88)	0.07 [-0.00, 0.14]
$d_{between}$, 95% *CI*	-	0.07 [-0.02, 0.16]	0.06 [-0.03, 0.14]	-0.03 [-0.12, 0.07]	
P value	-	.11	.22	.57	
High-risk (*n*=205)[2]					
PROTECT$_{training}$	32.65 (16.88)	31.49 (17.18)	31.37 (17.09)	29.36 (16.71)	-0.20 [-0.38, -0.01]
PROTECT$_{info}$	32.65 (16.25)	30.81 (15.44)	29.74 (15.16)	31.14 (16.57)	-0.09 [-0.29, 0.11]
$d_{between}$, 95% *CI*	-	0.04 [-0.21, 0.29]	0.10 [-0.16, 0.36]	-0.11 [-0.37, 0.15]	
P value	-	.75	.45	.42	
Not at risk (*n*=1588)[1]					
PROTECT$_{training}$	22.38 (12.72)	22.59 (13.35)	22.64 (14.10)	23.47 (14.07)	0.08 [0.00, 0.15]
PROTECT$_{info}$	22.38 (13.15)	21.54 (13.15)	21.96 (13.29)	23.68 (14.40)	0.09 [0.02, 0.16]
$d_{between}$, 95% *CI*	-	0.08 [-0.02, 0.18]	0.05 [-0.04, 0.14]	-0.01 [-0.11, 0.08]	
P value	-	.11	.29	.76	
School anxiety symptoms, FSSC-R					
Total sample (*N*=1793)[1]					
PROTECT$_{training}$	7.02 (4.21)	6.59 (4.31)	6.14 (4.48)	6.24 (4.50)	-0.17 [-0.24, -0.11]
PROTECT$_{info}$	7.02 (4.09)	6.36 (4.26)	6.08 (4.38)	6.30 (4.46)	-0.16 [-0.23, -0.09]
$d_{between}$, 95% *CI*	-	0.06 [-0.03, 0.14]	0.01 [-0.07, 0.10]	-0.01 [-0.11, 0.08]	
P value	-	.20	.75	.80	
High-risk (*n*=205)[2]					
PROTECT$_{training}$	9.24 (4.46)	8.30 (4.76)	7.84 (5.17)	8.21 (5.01)	-0.22 [-0.41, -0.02]
PROTECT$_{info}$	9.24 (4.19)	8.49 (4.46)	7.92 (4.12)	8.28 (4.52)	-0.20 [-0.39, -0.02]
$d_{between}$, 95% *CI*	-	-0.04 [-0.29, 0.21]	-0.02 [-0.27, 0.24]	-0.01 [-0.27, 0.25]	
P value	-	.74	.90	.92	
Not at risk (*n*=1588)[1]					
PROTECT$_{training}$	6.74 (4.09)	6.37 (4.20)	5.92 (4.33)	5.99 (4.36)	-0.17 [-0.24, -0.10]
PROTECT$_{info}$	6.74 (3.98)	6.08 (4.15)	5.85 (4.36)	6.04 (4.38)	-0.16 [-0.23, -0.08]
$d_{between}$, 95% *CI*	-	0.07 [-0.02, 0.16]	0.02 [-0.07, 0.11]	-0.01 [-0.11, 0.09]	
P value	-	.13	.70	.80	

(*To be continued*)

© 2026 Lindenberg K et al. *JAMA Network Open.*

eTable 4. (*Continued*)

	Pretest	Posttest	4 months	12 months	d_{within}, 95% *CI* (Pretest vs 12 months)
	M(*SD*)	*M*(*SD*)	*M*(*SD*)	*M*(*SD*)	
ADHD symptoms, Conners-3					
Total sample (*N*=1793)[1]					
$PROTECT_{training}$	8.20 (5.12)	8.25 (5.22)	8.22 (5.67)	8.92 (6.07)	0.12 [0.04, 0.19]
$PROTECT_{info}$	8.20 (5.05)	8.22 (5.16)	8.22 (5.52)	9.23 (6.40)	0.16 [0.09, 0.24]
$d_{between}$, 95% *CI*	-	0.01 [-0.10, 0.11]	-0.00 [-0.10, 0.10]	-0.05 [-0.14, 0.04]	
P value	-	.92	1.00	.27	
High-risk (*n*=205)[2]					
$PROTECT_{training}$	12.73 (5.70)	11.90 (5.56)	10.75 (6.32)	11.95 (5.86)	-0.12 [-0.35, 0.10]
$PROTECT_{info}$	12.73 (5.66)	11.87 (5.48)	12.09 (6.18)	11.48 (6.65)	-0.20 [-0.42, 0.01]
$d_{between}$, 95% *CI*	-	0.01 [-0.30, 0.31]	-0.21 [-0.49, 0.06]	0.08 [-0.21, 0.36]	
P value	-	.97	.13	.60	
Not at risk (*n*=1588)[1]					
$PROTECT_{training}$	7.61 (4.78)	7.78 (5.00)	7.89 (5.51)	8.53 (6.00)	0.15 [0.07, 0.23]
$PROTECT_{info}$	7.61 (4.58)	7.75 (4.90)	7.72 (5.20)	8.93 (6.30)	0.22 [0.14, 0.29]
$d_{between}$, 95% *CI*	-	0.01 [-0.11, 0.12]	0.03 [-0.07, 0.14]	-0.07 [-0.16, 0.03]	
P value	-	.93	.54	.17	
Procrastination, PFS-4					
Total sample (*N*=1793)[1]					
$PROTECT_{training}$	8.96 (3.72)	9.03 (3.83)	8.91 (3.99)	9.80 (4.13)	0.20 [0.13, 0.26]
$PROTECT_{info}$	8.96 (3.90)	8.95 (4.03)	9.06 (4.11)	10.00 (4.44)	0.24 [0.17, 0.31]
$d_{between}$, 95% *CI*	-	0.02 [-0.07, 0.10]	-0.04 [-0.12, 0.05]	-0.05 [-0.14, 0.05]	
P value	-	.68	.42	.34	
High-risk (*n*=205)[2]					
$PROTECT_{training}$	12.05 (4.26)	11.69 (4.11)	10.94 (4.30)	11.97 (4.26)	-0.02 [-0.23, 0.20]
$PROTECT_{info}$	12.05 (4.07)	11.65 (4.03)	11.30 (4.32)	12.30 (4.28)	0.06 [-0.16, 0.28]
$d_{between}$, 95% *CI*	-	0.01 [-0.26, 0.28]	-0.08 [-0.35, 0.18]	-0.08 [-0.37, 0.21]	
P value	-	.94	.54	.60	
Not at risk (*n*=1588)[1]					
$PROTECT_{training}$	8.56 (3.50)	8.67 (3.67)	8.65 (3.88)	9.52 (4.04)	0.23 [0.16, 0.30]
$PROTECT_{info}$	8.56 (3.61)	8.61 (3.87)	8.77 (3.97)	9.70 (4.35)	0.27 [0.20, 0.35]
$d_{between}$, 95% *CI*	-	0.02 [-0.08, 0.11]	-0.03 [-0.12, 0.06]	-0.04 [-0.14, 0.05]	
P value	-	.72	.50	.37	
Emotion dysregulation, DERS-SF					
Total sample (*N*=1793)[1]					
$PROTECT_{training}$	38.88 (12.98)	38.98 (13.54)	39.15 (14.43)	39.72 (14.22)	0.06 [-0.00, 0.12]
$PROTECT_{info}$	38.88 (13.30)	38.19 (13.46)	39.09 (14.43)	38.89 (13.50)	0.00 [-0.07, 0.07]
$d_{between}$, 95% *CI*	-	0.06 [-0.02, 0.14]	0.00 [-0.07, 0.08]	0.06 [-0.03, 0.15]	
P value	-	.17	.91	.17	
High-risk (*n*=205)[2]					
$PROTECT_{training}$	49.38 (14.42)	48.51 (15.87)	47.55 (15.95)	46.13 (16.08)	-0.20 [-0.39, -0.02]
$PROTECT_{info}$	49.38 (15.02)	48.48 (15.16)	47.50 (15.77)	47.57 (15.72)	-0.11 [-0.30, 0.07]
$d_{between}$, 95% *CI*	-	0.00 [-0.24, 0.24]	0.00 [-0.23, 0.24]	-0.09 [-0.34, 0.16]	
P value	-	.99	.98	.48	
Not at risk (*n*=1588)[1]					
$PROTECT_{training}$	37.51 (12.11)	37.72 (12.66)	38.05 (13.83)	38.87 (13.73)	0.10 [0.03, 0.17]
$PROTECT_{info}$	37.51 (12.41)	36.85 (12.61)	37.99 (13.87)	37.76 (12.75)	0.02 [-0.05, 0.09]
$d_{between}$, 95% *CI*	-	0.07 [-0.02, 0.16]	0.00 [-0.08, 0.09]	0.08 [-0.01, 0.17]	
P value	-	.14	.92	.07	

Note. [1]3-level model (observations in participants in classes), [2]2-level model (observations in participants). Estimated marginal means were derived from hierarchical linear models including time and the group × time interaction as fixed effects. Time was treated as a factor (0, 1, 4, and 12 months). CIUS, Compulsive Internet Use Scale, GAT, Gaming Attitude Test,

© 2026 Lindenberg K et al. *JAMA Network Open.*

GDT, Gaming Disorder Test, IGD-9, Internet Gaming Disorder Scale, SMDS, Social Media Disorder Scale, SDQ, Strength and Difficulties Questionnaire, KIDSCREEN-27, 27-item version of the KIDSCREEN questionnaire, WHO-5, WHO well-being index, DesTeen, depression screener for teenagers, SIAS, Social Interaction Anxiety Scale, FSSC-R, Fear Survey Schedule for Children – Revised, Conners-3, Conners 3rd Edition, PFS-4, 4-item version of the Procrastination Questionnaire for Students, DERS-SF, Difficulties in Emotion Regulation Scale – Short Form.

© 2026 Lindenberg K et al. *JAMA Network Open.*

eTable 5. Responder Analysis. Frequencies of Reliable Improvement From Pretest to 12-Months Follow-Up on Primary (Observed Data)

	Reliable improvement, *n*(%)	No reliable improvement, *n*(%)	Test statistic
Total sample (*n*=1095)			
$PROTECT_{training}$	71 (12.7)	486 (87.3)	$\chi^2(1) = 2.01$, $P = .16$
$PROTECT_{info}$	53 (9.9)	485 (90.1)	
High- risk (*n*=113)			
$PROTECT_{training}$	26 (46.4)	30 (53.6)	$\chi^2(1) = 0.42$, $P = .52$
$PROTECT_{info}$	22 (38.6)	35 (61.4)	
Not at risk (*n*=982)			
$PROTECT_{training}$	45 (9.0)	456 (91.0)	$\chi^2(1) = 1.87$, $P = .17$
$PROTECT_{info}$	31 (6.4)	450 (93.6)	

Note. Reliable improvement was defined as RCI ≤ -1.96. Primary outcome is the modified version of the video game dependency scale at 12 months to capture Gaming and unspecified Internet use disorders.

© 2026 Lindenberg K et al. *JAMA Network Open.*

eTable 6. Negative Effects. Frequencies of Reliable Deterioration From Pretest to 12-Months Follow-Up on Primary Outcome (Observed Data)

	Reliable deterioration, *n*(%)	No reliable deterioration, *n*(%)	Test statistic
Total sample (*n*=1095)			
$PROTECT_{training}$	44 (7.9)	513 (92.1)	$\chi^2(1) = 0.37$, $P = .54$
$PROTECT_{info}$	49 (9.1)	489 (90.9)	
High-risk (*n*=113)[1]			
$PROTECT_{training}$	2 (3.6)	54 (96.4)	$\chi^2 = 1.31$, $P = .44$
$PROTECT_{info}$	5 (8.8)	52 (91.2)	
Not at risk (*n*=982)			
$PROTECT_{training}$	42 (8.4)	459 (91.6)	$\chi^2(1) = 0.10$, $P = .76$
$PROTECT_{info}$	44 (9.1)	437 (90.9)	

Note. Reliable deterioration was defined as RCI ≥ 1.96. Primary outcome is the modified version of the video game dependency scale at 12 months to capture Gaming and unspecified Internet use disorders. [1]Chi-squared test with simulated p-value (based on 2000 replicates).

© 2026 Lindenberg K et al. *JAMA Network Open.*

eTable 7. Adherence. Descriptive Statistics of Adherence Ratings

	PROTECTtraining	PROTECTinfo
	M(*SD*)	*M*(*SD*)
Module 1	1.79 (0.19)[1]	1.84 (0.17)[2]
Module 2	1.84 (0.20)[2]	1.77 (0.22)[3]
Module 3	1.77 (0.22)[4]	1.81 (0.22)[4]
Module 4	1.72 (0.26)[2]	1.81 (0.22)[2]

Note. [1]n = 89, [2]n = 87, [3]n = 85, [4]n = 84 half-class groups. Total mean scores across items reflecting adherence to the specified intervention elements, aggregated across half-class groups, are presented. Items scaled (2) "completely adherent", (1) "partially adherent", and (0) "not adherent".

© 2026 Lindenberg K et al. *JAMA Network Open.*

eReferences.

1. Lindenberg K, Kindt S, Szász-Janocha C. *Internet Addiction in Adolescents*. Springer International Publishing; 2020.
2. Rehbein F, Kliem S, Baier D, Mößle T, Petry NM. Prevalence of Internet gaming disorder in German adolescents: diagnostic contribution of the nine DSM-5 criteria in a state-wide representative sample. *Addiction*. 2015;110(5):842-851. doi:10.1111/add.12849
3. Rehbein F, Baier D, Kleimann M, Mößle T. *Computerspielabhängigkeitsskala (CSAS): Ein Verfahren Zur Erfassung Der Internet Gaming Disorder Nach DSM-5*. Hogrefe; 2015.
4. Lindenberg K, Kindt S, Szász-Janocha C. Effectiveness of Cognitive Behavioral Therapy-Based Intervention in Preventing Gaming Disorder and Unspecified Internet Use Disorder in Adolescents: A Cluster Randomized Clinical Trial. *JAMA Netw Open*. 2022;5(2):e2148995. doi:10.1001/jamanetworkopen.2021.48995
5. Meerkerk G-J, van den Eijnden RJJM, Vermulst AA, Garretsen HFL. The Compulsive Internet Use Scale (CIUS): some psychometric properties. *Cyberpsychol Behav*. 2009;12(1):1-6. doi:10.1089/cpb.2008.0181
6. Besser B, Rumpf H-J, Bischof A, Meerkerk G-J, Higuchi S, Bischof G. Internet-Related Disorders: Development of the Short Compulsive Internet Use Scale. *Cyberpsychol Behav Soc Netw*. 2017;20(11):709-717. doi:10.1089/cyber.2017.0260
7. Lemmens JS, Valkenburg PM, Gentile DA. The Internet Gaming Disorder Scale. *Psychol Assess*. 2015;27(2):567-582. doi:10.1037/pas0000062
8. Pontes HM, Schivinski B, Sindermann C, et al. Measurement and Conceptualization of Gaming Disorder According to the World Health Organization Framework: the Development of the Gaming Disorder Test. *Int J Ment Health Addiction*. 2021;19(2):508-528. doi:10.1007/s11469-019-00088-z
9. Lindenberg K, Hofmann S. Überprüfung der Effekte dissonanzbasierter Primärprävention von Internet- und Computerspielabhängigkeit in der Jahrgangsstufe 5 auf die Einstellung zum Gaming: Eine Pilotstudie. *Z Kinder Jugendpsychiatr Psychother*. 2021;50(1):25-36. doi:10.1024/1422-4917/a000814
10. van den Eijnden RJ, Lemmens JS, Valkenburg PM. The Social Media Disorder Scale. *Computers in Human Behavior*. 2016;61:478-487. doi:10.1016/j.chb.2016.03.038
11. Goodman R, Meltzer H, Bailey V. The Strengths and Difficulties Questionnaire: a pilot study on the validity of the self-report version. *Int Rev Psychiatry*. 2003;15(1-2):173-177. doi:10.1080/0954026021000046137
12. Lidzba K, Christiansen H, Drechsler R. Conners-3. Conners Skalen zu Aufmerksamkeit und Verhalten – 3. Deutschsprachige Adaptation der Conners 3rd EditionTM (Conners 3TM) von C. Keith Conners. 2013. https://www.zora.uzh.ch/id/eprint/74782/
13. Pietsch K, Allgaier A-K, Frühe B, et al. Screening for depression in adolescent paediatric patients: validity of the new Depression Screener for Teenagers (DesTeen). *J Affect Disord*. 2011;133(1-2):69-75. doi:10.1016/j.jad.2011.03.026
14. Mattick RP, Clarke JC. Development and validation of measures of social phobia scrutiny fear and social interaction anxiety. *Behav Res Ther*. 1998;36(4):455-470. doi:10.1016/s0005-7967(97)10031-6
15. Döpfner M, Schnabel M, Goletz H, Ollendick T. *Phobiefragebogen Für Kinder Und Jugendliche*. Hogrefe; 2006.
16. Kaufman EA, Xia M, Fosco G, Yaptangco M, Skidmore CR, Crowell SE. The Difficulties in Emotion Regulation Scale Short Form (DERS-SF): Validation and Replication in Adolescent and Adult Samples. *J Psychopathol Behav Assess*. 2016;38(3):443-455. doi:10.1007/s10862-015-9529-3
17. Glöckner-Rist A, Engberding M, Höcker A, Rist F. *Prokrastinationsfragebogen für Studierende (PFS). In A. Glöckner-Rist (Ed.), Zusammenstellung sozialwissenschaftlicher Items und Skalen. ZIS Version 13.00. Bonn: GESIS; 2009*; 2009.

© 2026 Lindenberg K et al. *JAMA Network Open.*

18. Ravens-Sieberer U, Auquier P, Erhart M, et al. The KIDSCREEN-27 quality of life measure for children and adolescents: psychometric results from a cross-cultural survey in 13 European countries. *Qual Life Res*. 2007;16(8):1347-1356. doi:10.1007/s11136-007-9240-2
19. Topp CW, Østergaard SD, Søndergaard S, Bech P. The WHO-5 Well-Being Index: a systematic review of the literature. *Psychother Psychosom*. 2015;84(3):167-176. doi:10.1159/000376585
20. Kupper K, Krampen D, Rammstedt B, Rohrmann S. Kurzversion des Big Five Inventory für Kinder und Jugendliche (BFI-K KJ). *Diagnostica*. 2019;65(2):86-96. doi:10.1026/0012-1924/a000216
21. Saunders JB, Aasland OG, Babor TF, La Fuente JR de, Grant M. Development of the Alcohol Use Disorders Identification Test (AUDIT): WHO Collaborative Project on Early Detection of Persons with Harmful Alcohol Consumption--II. *Addiction*. 1993;88(6):791-804. doi:10.1111/j.1360-0443.1993.tb02093.x
22. Legleye S, Karila L, Beck F, Reynaud M. Validation of the CAST, a general population Cannabis Abuse Screening Test. *Journal of Substance Use*. 2007;12(4):233-242. doi:10.1080/14659890701476532
23. Johnson EE, Hamer R, Nora RM, Tan B, Eisenstein N, Engelhart C. The Lie/Bet Questionnaire for screening pathological gamblers. *Psychol Rep*. 1997;80(1):83-88. doi:10.2466/pr0.1997.80.1.83
24. Müller A, Trotzke P, Mitchell JE, Zwaan M de, Brand M. The Pathological Buying Screener: Development and Psychometric Properties of a New Screening Instrument for the Assessment of Pathological Buying Symptoms. *PLoS One*. 2015;10(10):e0141094. doi:10.1371/journal.pone.0141094
25. Coffman CJ, Edelman D, Woolson RF. To condition or not condition? Analysing 'change' in longitudinal randomised controlled trials. *BMJ Open*. 2016;6(12):e013096. doi:10.1136/bmjopen-2016-013096
26. Harrer M, Miguel C, Luo Y, et al. Standardized effect sizes are far from "Standardized": A primer and empirical illustration in depression psychotherapy meta-analyses. *PLOS Ment Health*. 2025;2(7):e0000347. doi:10.1371/journal.pmen.0000347
27. Jacobson NS, Truax P. Clinical significance: a statistical approach to defining meaningful change in psychotherapy research. *J Consult Clin Psychol*. 1991;59(1):12-19. doi:10.1037/0022-006X.59.1.12
28. Robitzsch A, Grund S. *CRAN: Contributed Packages*. 2014.

© 2026 Lindenberg K et al. *JAMA Network Open.*
