# Supplementary material for: Cognitive Behavioral Therapy vs Media Literacy for Prevention of Gaming Disorder and Unspecified Internet Use Disorder: A Randomized Clinical Trial
Source: JAMA Netw Open. 2026 Sep 4;9(9):e2631942. doi: 10.1001/jamanetworkopen.2026.31942 (PMC13545672; doi:10.1001/jamanetworkopen.2026.31942)
Supplement: Supplementary file 3 [file jamanetwopen-e2631942-s003.pdf]

Data Sharing Statement

Lindenberg. Cognitive Behavioral Therapy vs Media Literacy for Prevention of Gaming Disorder and Unspecified Internet Use Disorder. *JAMA Netw Open*. Published September 04, 2026. doi:10.1001/jamanetworkopen.2026.31942

Data
Additional Information: German register for clinical trials, https://drks.de/search/en/trial/DRKS00033989, trial registry number DRKS00033989
Data available: Yes
Data types: Deidentified participant data
How to access data: katajun.lindenberg@psychologie.uni-heidelberg.de
When available: With publication

Supporting Documents
Document types: Statistical/analytic code, Other (please specify)
Additional Information: Manual
How to access documents: Statistical analysis code is available at doi:10.23668/PSYCHARCHIVES.22350. Complete HTML output and PROTECTinfo Manual (in German language) are available at doi:10.23668/PSYCHARCHIVES.22351. PROTECTtraining (in English language) is available in Lindenberg et al., 2020. Please contact the corresponding authors for international translation
When available: With publication

Additional Information
Who can access the data: scientisis
Types of analyses: Data sharing. Anonymous data collected and presented in this study will be made available upon reasonable request after publication of this article. Statistical analysis code is available at doi:10.23668/PSYCHARCHIVES.22350. Requests for data will be reviewed by the study investigators and shared under a formal data use agreement. Data can be requested by contacting the corresponding author. Manuals for the two tested behavioral interventions are publicly available. PROTECTtraining (in English language) is available in Lindenberg et al., 2020. Please contact the corresponding authors for international translations. PROTECTinfo is available in German language at doi:10.23668/PSYCHARCHIVES.22351
Mechanisms of data availability: see above
